# Expression and Regulation of Deubiquitinase-Resistant, Unanchored Ubiquitin Chains in *Drosophila*

**DOI:** 10.1038/s41598-018-26364-x

**Published:** 2018-05-31

**Authors:** Jessica R. Blount, Kozeta Libohova, Gregory B. Marsh, Joanna R. Sutton, Sokol V. Todi

**Affiliations:** 1 Department of Pharmacology, Wayne State University School of Medicine, Detroit, MI USA; 2 Department of Neurology, Wayne State University School of Medicine, Detroit, MI USA

## Abstract

The modifier protein, ubiquitin (Ub) regulates various cellular pathways by controlling the fate of substrates to which it is conjugated. Ub moieties are also conjugated to each other, forming chains of various topologies. In cells, poly-Ub is attached to proteins and also exists in unanchored form. Accumulation of unanchored poly-Ub is thought to be harmful and quickly dispersed through dismantling by deubiquitinases (DUBs). We wondered whether disassembly by DUBs is necessary to control unanchored Ub chains *in vivo*. We generated *Drosophila melanogaster* lines that express Ub chains non-cleavable into mono-Ub by DUBs. These chains are rapidly modified with different linkages and represent various types of unanchored species. We found that unanchored poly-Ub is not devastating in *Drosophila*, under normal conditions or during stress. The DUB-resistant, free Ub chains are degraded by the proteasome, at least in part through the assistance of VCP and its cofactor, p47. Also, unanchored poly-Ub that cannot be cleaved by DUBs can be conjugated *en bloc*, *in vivo*. Our results indicate that unanchored poly-Ub species need not be intrinsically toxic; they can be controlled independently of DUB-based disassembly by being degraded, or through conjugation onto other proteins.

## Introduction

Posttranslational modification of proteins by ubiquitin (Ub) controls most cellular processes^1–3^. This modification adjusts how a protein interacts with cellular constituents, because Ub presents additional interaction interfaces^2,4,5^. Ubiquitination regulates protein localization, activity, function and half-life^2,6^. Addition and removal of Ub from specific proteins is vital and is implicated in human diseases^1–3,6^. Ubiquitination involves the covalent attachment of Ub to—most commonly—lysine residues of substrate proteins through the coordinated action of a Ub activating enzyme (E1), a Ub conjugating enzyme (E2) and a Ub ligase (E3). Since Ub itself contains seven lysines, different Ub chains can be formed by the attachment of one Ub to another at different lysine residues; head-to-tail/linear chains, chains with mixed-linkages and branched chains are also generated^2,7,8^. Arguably the best known chain is K48-linked poly-Ub, which targets proteins for proteasomal degradation^9^. Other types of chains also exist in cells^2,3,10–13^.

Similar to other types of posttranslational modification, ubiquitination is reversible. Ub removal is accomplished by deubiquitinases (DUBs) and serves multiple roles: 1) editing chains to reduce errors; 2) bringing a molecular process to an end by, for example, disassembling a protein complex that merged as a result of a member’s ubiquitination; 3) recycling mono-Ub; and 4) disassembling unanchored/free poly-Ub^3,5,6,14^. Unanchored poly-Ub results from *en bloc* cleavage of chains from substrates. Free chains function in autophagy-dependent processes, immune system-related steps and during DNA stress; unanchored poly-Ub chains are also synthesized anew^3,5,10,11,15–21^.

Unanchored poly-Ub is thought to be quickly disassembled by DUBs^2,5,6,22^. Accumulation of unanchored chains is proposed to be toxic by perturbing Ub-dependent processes. For instance, they may titrate out binding of ubiquitinated proteins to the proteasome^1,15–17,23–26^. To the best of our knowledge, there are no published reports that directly examine unanchored poly-Ub species in an intact organism to address questions such as: what happens to unanchored chains if DUBs cannot dismantle them? Are unanchored poly-Ub species inherently toxic *in vivo*?

We generated poly-Ub transgenes that are not cleavable by DUBs and function as unanchored species. Their expression in *Drosophila melanogaster* is not lethal during development and adult flies tolerate the poly-Ub species well. These unanchored, non-cleavable poly-Ub are themselves decorated with Ub and regulated by the proteasome. We also found that these chains can be utilized *en bloc* without the need to be dismantled into mono-Ub. We propose that unanchored poly-Ub can be regulated independently of DUB-based disassembly and suggest a need to re-evaluate the extent of toxicity from free chains. Additionally, the new tools that we developed should help future work to pursue regulation of unanchored poly-Ub in non-canonical ways.

## Results

### Expression of unanchored poly-Ub in *Drosophila*

**Figure 1:**
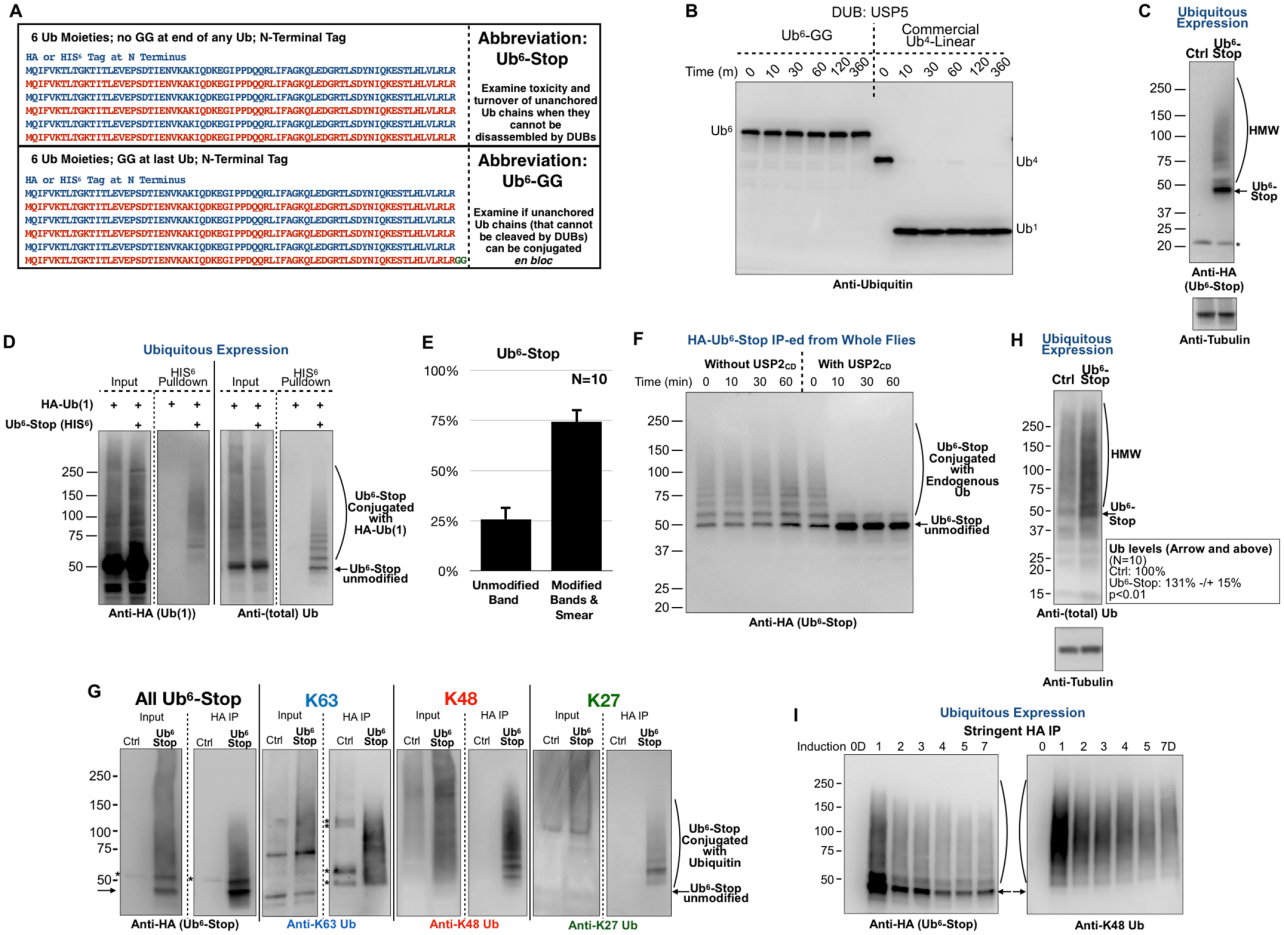
Non-cleavable poly-Ub in *Drosophila*. (**A**) Summary of poly-Ub constructs. (**B**) Recombinant, untagged Ub^6^ (1 µM) was incubated with USP5 (50 nM). See Methods for details. (**C**) Western blots of Ub^6^-Stop expressed in all tissues. Driver was sqh-Gal4. HMW: higher molecular weight. Asterisk: non-specific band. Flies were one day old. (**D**) Western blots from whole flies expressing the indicated transgenes, driven everywhere by sqh-Gal4. HIS^6^-pulldown of Ub^6^-Stop was conducted under denaturing conditions. Solid line separates blots from the same membrane probed with the indicated antibodies. Dotted lines separate lanes reorganized from the same membrane. Un-cropped blots are in Supplemental Figures. (**E**) Histograms summarizing Ub^6^-Stop that is unmodified (non-conjugated band) vs. modified (conjugated bands and upper smear) in blots in panels (**C**,**F**,**G**) and other, similar experiments. N = 10 independent repeats. Means −/+SD. Driver: sqh-Gal4. (**F**) Deubiquitination reaction of Ub^6^-Stop isolated from flies and incubated in the absence or presence of the catalytic domain of recombinant USP2 (USP2_CD_). See Methods for details. (**G**) Western blots from denature/renature IPs to examine ubiquitination of HA-tagged Ub^6^-Stop by the indicated linkages. Solid lines indicate that membrane was loaded multiple times with the same samples, same amounts, cut and probed simultaneously, as shown, to eliminate cross-contamination issues from stripping and re-probing the same membrane. Asterisks: non-specific bands that we observe consistently with the respective antibodies. Dotted lines separate lanes cropped from the same membrane. Un-cropped blots are shown in Supplemental Figures. (**H**) Western blots of whole, adult flies expressing Ub^6^-Stop in all tissues (driver was sqh-Gal4), and loaded to probe all Ub species. HMW: higher molecular weight. Box on the right summarizes quantification of signal from the adjacent blots and other, independent experiments, normalized to respective loading controls, with Ctrl. lanes set to 100%. P value is from two-tailed Student’s t-test. Signal quantified encompassed the band at the arrow all the way at the top of the gel. I) Western blots of stringent purification of HA-Ub^6^-Stop driven by tubulin-Gal4-GS, which is dependent on RU486 to induce expression of Ub^6^-Stop in all tissues; see main text for details. Arrows: unmodified Ub^6^-Stop. Curved lines: higher molecular weight species consistent with ubiquitinated Ub^6^-Stop. For panels (B–D,F–I), results are representative of experiments conducted independently at least thrice, with similar results. In all panels with *Drosophila* data, flies were heterozygous for driver and Ub^6^.

We devised a strategy to express in *Drosophila* unanchored poly-Ub that cannot be dismantled by DUBs. While this approach introduces exogenous Ub, we reasoned that our plan would directly examine unanchored chains that cannot be removed through deubiquitination. We designed two chains that consist of six Ub in tandem and lack internal “GG” motifs that are necessary for isopeptide bond formation and their dismantling by DUBs (Fig. 1A). The first (Ub^6^-Stop) cannot be cleaved by DUBs and lacks a terminal “GG”, meaning that it cannot itself be conjugated onto other proteins. The second version is conjugatable; it does not have internal “GG” motifs, but contains a “GG” at the end (Ub^6^-GG). We tested two DUBs for their ability to cleave these chains. As shown in Fig. 1B and Supplemental Fig. 1, the chains we generated are not cleaved *in vitro* by USP5. USP5 rapidly cleaves all types of chains *in vitro*, including linear chains^27^ and can also cleave chains with a free C-terminal “GG” or with an occupied “GG” (i.e. anchored chain)^2,22,27–29^. Figure 1F shows that another DUB, USP2, is also unable to cleave Ub^6^-Stop into single Ub. We began our studies with the non-cleavable, non-conjugatable Ub^6^-Stop. We should note that utilizing a linear chain is currently the only way to ensure efficient and robust production of non-cleavable poly-Ub in an intact organism. Importantly, as we describe below, these linear chains are quickly modified with different linkages to form various topologies.

We generated *Drosophila* lines that utilize the Gal4-UAS system^30,31^ to express Ub^6^-Stop. We began by expressing Ub^6^-Stop throughout the fly, using the driver sqh-Gal4, which expresses in all tissues, during development and in adults^32–37^. As shown in Fig. 1C, Ub^6^-Stop migrates as a number of species on western blots. The lowermost band is the expected, unmodified version of the protein, whereas the higher species are most likely posttranslationally modified. Based on results from stringent immunopurifications (IPs) with a denature/renature step, higher species consist of ubiquitinated versions of Ub^6^-Stop. We first co-expressed a mono-Ub transgene that is HA-tagged alongside Ub^6^-Stop that is HIS^6^ tagged, then precipitated HIS^6^-Ub^6^-Stop and examined its labeling by the mono-Ub. As shown in Fig. 1D, Ub^6^-Stop heavy molecular weight species, but not the unmodified form, are labelled by the antibody that detects the mono-Ub we introduced. This finding indicates that the Ub^6^-Stop linear chain is itself posttranslationally modified by Ub. Based on the quantification of Ub^6^-Stop conjugated and non-conjugated bands and smears, ~75% of the total Ub^6^-Stop is modified (Fig. 1E).

To additionally confirm that higher molecular weight Ub^6^-Stop species are ubiquitinated, we subjected Ub^6^-Stop from flies to the catalytic domain of USP2 (USP2_CD_). Within 10 minutes, the higher molecular weight species collapse to the expected, unmodified Ub^6^-Stop in the presence of USP2_CD_ (Fig. 1F). A band immediately above Ub^6^-Stop remains stable. Based on our stringent IPs (Fig. 1D,G), this is most likely mono-ubiquitinated Ub^6^-Stop. USP2_CD_ might be unable to remove this modification. Also, we cannot formally discount the possibility of another type of posttranslational modification of this Ub^6^-Stop band. This species notwithstanding, our results demonstrate that Ub^6^-Stop is itself ubiquitinated in the fly.

To get a glimpse at the type of Ub linkages attached onto Ub^6^-Stop, we conducted additional, stringent IPs and probed Ub^6^-Stop with antibodies against K27, K48 and K63 linkages. We found that Ub^6^-Stop is modified with K27, K48 and K63 linkages (Fig. 1G). Ub^6^-Stop itself is essentially a linear chain; the fact that there are K27, K48 and K63 Ub-Ub conjugates on it means that the higher molecular weight species of Ub^6^-Stop constitute branched chains with various Ub-Ub linkages. These types of poly-Ub species exist in cells^7,8^. Our analysis does not distinguish whether a specific branch of Ub moieties added onto Ub^6^-Stop comprises the same type or different types of linkages. Based on these biochemical data, we conclude that higher molecular weight species of Ub^6^-Stop contain branched chains and various Ub-Ub linkages.

When compared to the rest of Ub in the fly by western blotting, Ub^6^-Stop species are abundant in* Drosophila*. We observe the unmodified band of Ub^6^-Stop, as well as an overall darkening of the Ub smear above it (Fig. 1H), which most likely consists of Ub^6^-Stop conjugated with endogenous Ub. Quantification of these data shows a statistically significant increase in Ub signal in the presence of Ub^6^-Stop, when expressed in all tissues (Fig. 1H, quantification box). Thus, Ub^6^-Stop species appear to be prominently expressed.

To assess how quickly Ub^6^-Stop is ubiquitinated, we used the inducible, RU486-dependent ubiquitous driver, tubulin-Gal4-GS to drive the Ub^6^ construct. We raised flies in media without RU486. On day 1 as adults, flies were switched to RU486-food for 0–7 days. Ub^6^-Stop is rapidly produced (Fig. 1I). Within 24 hours of being placed into RU486-containing media, we observe prominent Ub^6^-Stop species, which reach an equilibrium over 3–4 days. This is most likely due to initial Ub^6^-Stop production followed by degradation as the process stabilizes.

**Figure 2:**
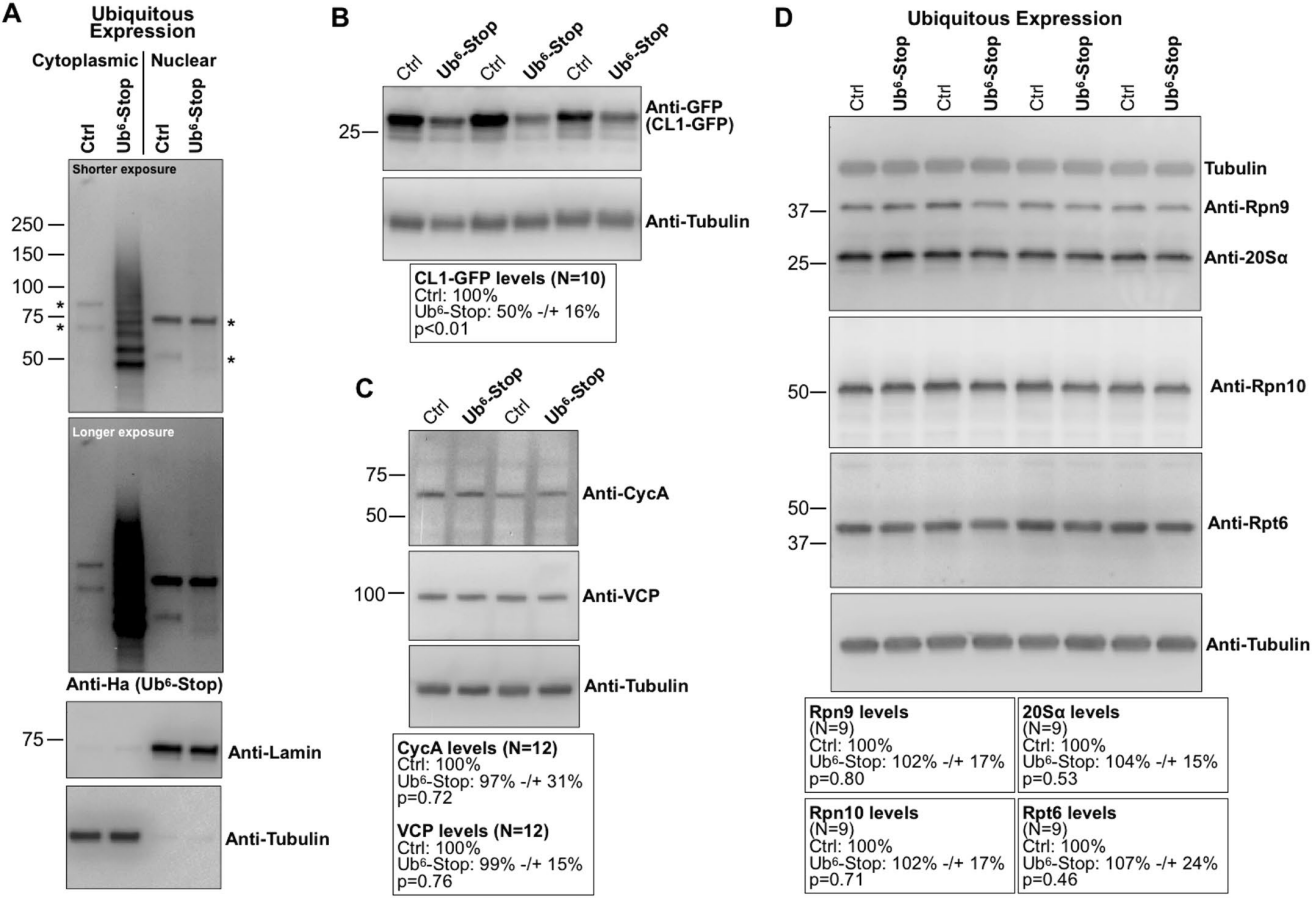
Ub^6^-Stop does not inhibit protein degradation or affect proteasome levels in *Drosophila*. (**A**) Western blots from cytoplasmic/nuclear fractionation of whole flies expressing Ub^6^-Stop in all tissues (Methods). Results are representative of experiments conducted independently at least three times, with similar results. Asterisks: non-specific signal. Driver was sqh-Gal4. Two different exposures are shown for Ub signal. (**B**,**C**) Western blots of the levels of the indicated proteins in the absence or presence of Ub^6^-Stop. Boxes underneath blots show quantification summaries of the levels of the respective proteins. Means −/+SD. Statistics: two-tailed Student’s t-tests. For (**B**) CL1-GFP was co-expressed alongside Ub^6^-Stop in fly eyes, driven by GMR-Gal4. Flies were one day old. Full blots are in Supplemental Figures. For (**C**) we probed one day old whole adults expressing Ub^6^-Stop in all tissues through sqh-Gal4. The different lanes in blots represent independent experimental repeats. (**D**) Levels of endogenous proteasome subunits when Ub^6^-Stop is expressed everywhere via sqh-Gal4. Flies were one day old. Boxes underneath blots show quantification summaries of the levels of the respective proteasome proteins. Means −/+SD. Statistics: two-tailed Student’s t-tests. The different lanes in all blots represent independent experimental repeats. Tubulin signal in top blot remains after stripping to probe with the antibodies indicated in that panel. In all panels, flies were heterozygous for driver and transgenes.

Next, we assessed the distribution of Ub^6^-Stop into major cellular sub-compartments. We found that these species are cytoplasmic (Fig. 2A). We also found that their expression does not impair proteasome-dependent degradation (Fig. 2B,C). We examined endogenous cyclin A, a proteasome substrate, and CL1-GFP, a reporter of proteasome activity in mammalian and fly systems; the CL1 degron targets GFP for ubiquitination and subsequent proteasome-dependent degradation^27,38–40^. Panels 2B and 2 C show that the levels of neither proteasome substrate increase in the presence of Ub^6^-Stop. In fact, we observed diminished levels of CL1-GFP with Ub^6^-Stop, indicating increased—not decreased—proteasome activity. The levels of cyclin A did not change in the presence of Ub^6^-Stop. We also blotted for VCP, whose expression is increased when proteasome function is reduced in the fly^41^ and did not find significant differences in the presence of Ub^6^-Stop (Fig. 2C). Lastly, endogenous proteasome subunits were not significantly impacted by the presence of Ub^6^-Stop (Fig. 2D). Results in Fig. 2B–D indicate that Ub^6^-Stop does not inhibit the activity of the proteasome *in vivo* and that its presence does not perturb levels of endogenous proteasome components.

### Expression of unanchored poly-Ub is not devastating in *Drosophila*

**Figure 3:**
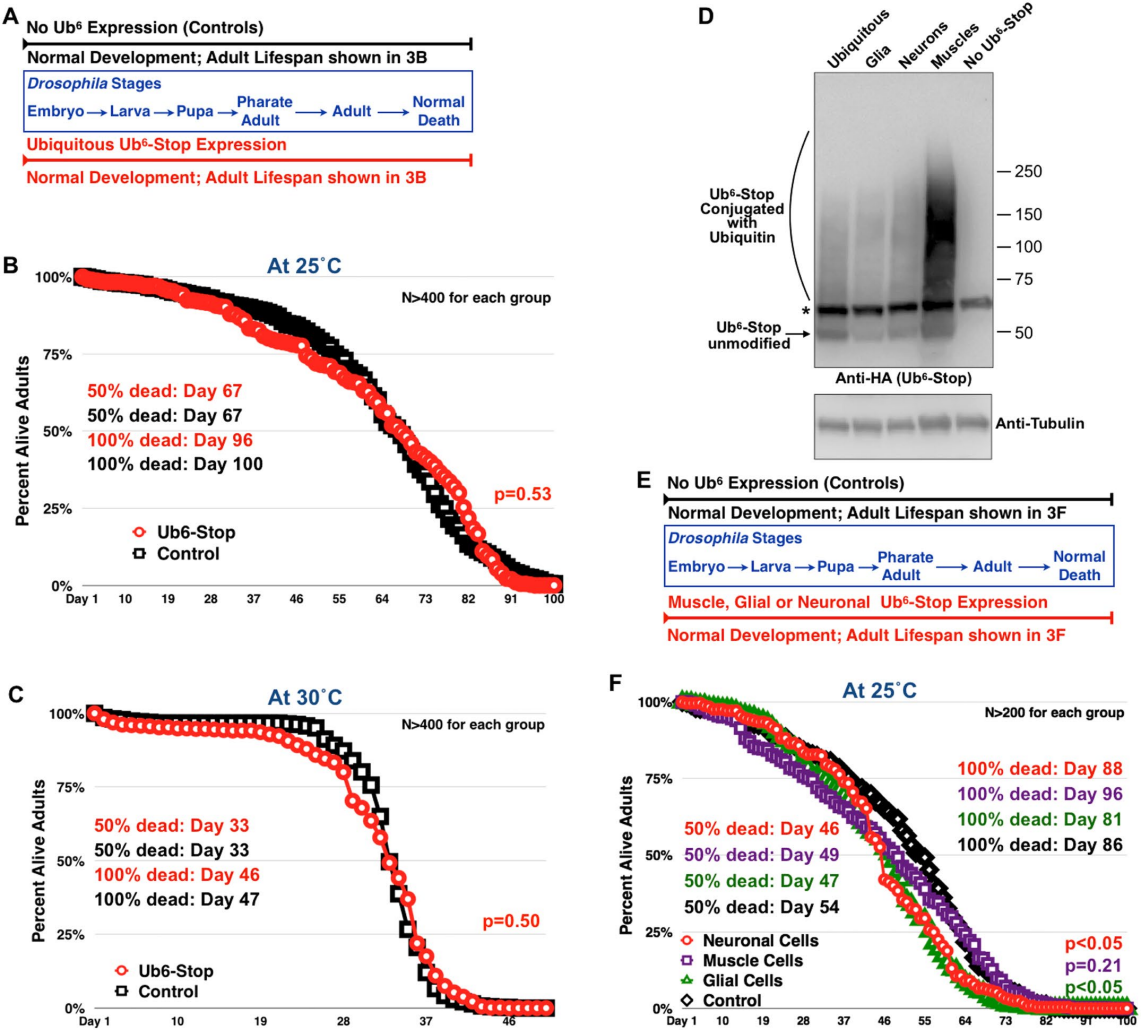
Expression of Ub^6^-Stop everywhere or in specific tissues is not lethal to *Drosophila*. (**A**) Summary of the effects of expression of Ub^6^-Stop throughout fly development. Driver was sqh-Gal4. Control: sqh-Gal4 on the genetic background used to generate the Ub^6^ flies. We monitored daily lethality at larval, pupal and pharate adult stages among groups and did not notice differences in their development. Little to no developmental lethality occurred in all crosses. We monitored at least 10 independent crosses, all at 25 °C. (**B**,**C**) Percent longevity of adult flies not expressing or expressing Ub^6^-Stop at the indicated temperatures. P values were calculated using log-rank (Mantel-Cox) tests. (**D**) Western blots showing the expression levels of Ub^6^-Stop in the indicated tissues. We used one day old, whole adult flies for all lysates. Results are representative of experiments conducted independently four or more times, with similar results. Asterisk: non-specific band. (**E**) Summary of lethality during development when Ub^6^-Stop was expressed in specific tissues, as in (**A**). We monitored lethality at larval, pupal and pharate adult stages. We did not notice differences in their development and little to no developmental lethality occurred in all crosses. We monitored at least 10 independent crosses, all at 25 °C. (**F**) Percent longevity of adult flies not expressing or expressing Ub^6^-Stop in the indicated fly tissues. Controls consisted of the respective drivers on the genetic background utilized to generate Ub^6^ flies, but without the transgene. P values were calculated using log-rank (Mantel-Cox) tests. In all panels, flies were heterozygous for driver and Ub^6^.

We examined lethality from unanchored chains in *Drosophila* by driving the expression of Ub^6^-Stop through sqh-Gal4. This is a highly robust driver that others and we have used with strong outcomes, such as early developmental lethality from knockdown of various genes and high toxicity from expression of various, mutant proteins^27,32–35,37,42–47^. *Drosophilae* undergo several developmental stages, none of which appears impacted by Ub^6^-Stop (Fig. 3A). When we tracked adult fly longevity in the absence or presence of Ub^6^-Stop, we again did not notice marked deviation between the two groups (Fig. 3B). This lack of a statistically significant difference in longevity in adult flies expressing Ub^6^-Stop was also noticeable when adults were stressed with heat. As shown in Fig. 3C, flies expressing Ub^6^-Stop and placed at 30 °C live similarly to their non-Ub^6^-Stop counterparts.

We subsequently expressed Ub^6^-Stop in select fly tissues—neurons, glia and muscle cells—using drivers common to the fly community. We selected this approach because tissue-specific drivers can express UAS transgenes more strongly in that particular tissue than ubiquitous drivers, as exemplified in Fig. 3D. Figure 3D shows expression of Ub^6^-Stop in different tissues; the muscle driver expresses this construct very highly. Developmental observations did not show marked differences in lethality from Ub^6^-Stop expressed in the tissues tested (Fig. 3E). When we examined adult fly longevity, 50% were dead a few days earlier than controls in the presence of Ub^6^-Stop (Fig. 3F). Comparison of the day when all flies were dead showed that with muscle expression, adults expressing Ub^6^-Stop persisted longer than controls. Statistical analyses of these results revealed a significant difference in the overall longevity of flies expressing Ub^6^-Stop in glial or neuronal cells compared to control flies: presence of Ub^6^-Stop in these tissues led to overall shorter lifespan. Still, this is a mild deviation. Muscle-specific expression did not lead to a statistically different lifespan compared to controls. Collectively, these data lead us to the conclusion that Ub^6^-Stop is not devastating overall.

Longevity studies with the ubiquitous driver (Fig. 3A–C) and tissue-specific drivers (Fig. 3D–F) were conducted at different times, maintained in different rooms and reared and aged in fly media prepared with different batches of primary ingredients. These factors most likely account for the overall shorter longevity of flies in Fig. 3F compared to 3B. However, control flies for each experiment (Fig. 3A,B,C,E,F) were collected, aged and observed side by side with the Ub^6^-Stop flies.

### Protein levels of Ub^6^-Stop depend on the proteasome

**Figure 4:**
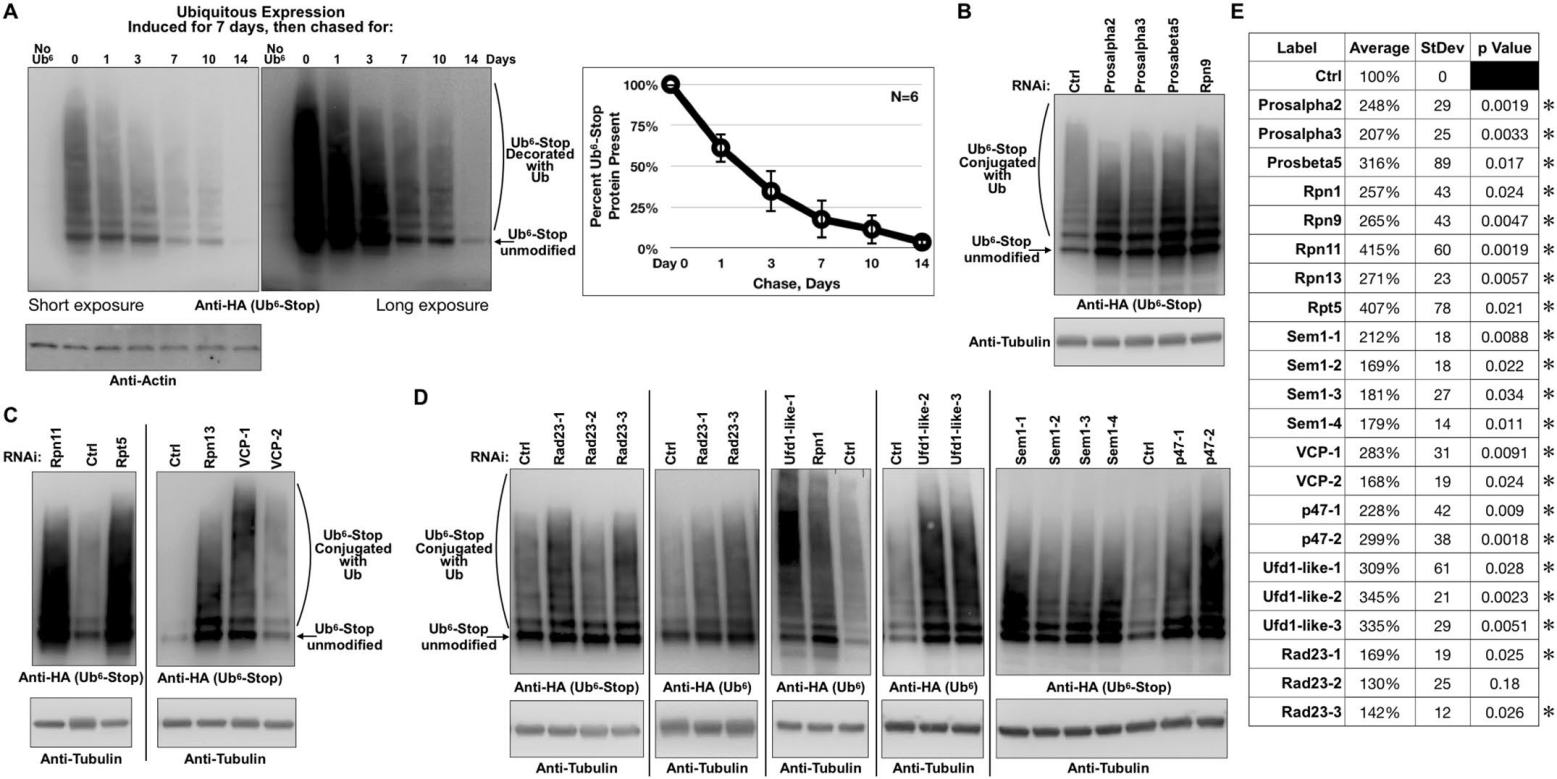
Ub^6^-Stop turnover in *Drosophila*. (**A**) Left: Western blots of whole fly lysates expressing Ub^6^-Stop via tubulin-Gal4-GS. Flies were reared in media without RU486. On day 1 as adults, they were switched to media with the inducer and allowed to produce Ub^6^-Stop for seven days. On day 7, flies were switched to media without RU486 to shut off Ub^6^-Stop production and whole flies were frozen on the indicated days. Right: quantification of the entire Ub^6^-Stop smear from the images on the left and other, independent experiments. Means −/+SD. N = 6 independent repeats. Two different exposures are shown for Ub signal. (**B–D**) Western blots from dissected fly heads expressing Ub^6^-Stop and the indicated RNAi lines. Numbers denote different RNAi lines. In all panels, flies were heterozygous for GMR-Gal4, Ub^6^-Stop and RNAi transgenes. Results are representative of experiments conducted independently three or more times with each RNAi line, with similar results. For Rad23 lines we are including results from two independent experiments, since the results are not as clear or consistent as with the other lines from experiment to experiment. Samples, gels and probes were conducted on different days. Samples in one membrane were collected and prepared on the same day. (**E**) Table summarizing quantification of images from blots in panels (**B–D**) and other, independent experiments. The entire Ub^6^-Stop signal was quantified. N is at least three for each RNAi line and controls. Signal from RNAi lines was normalized to its respective control. Means −/+SD. P values are from two-tailed Student’s t-tests comparing each RNAi to its own control (no RNAi). Asterisks: p < 0.05.

Since Ub^6^-Stop cannot be disassembled by DUBs, we wondered whether it can be regulated by the proteasome. First, we assessed its persistence *in vivo*. We again utilized the RU486-dependent, ubiquitous driver, tubulin-Gal4-GS. Flies that contained one copy each of the driver and Ub^6^-Stop transgenes were reared in food without RU486 until day 1 as adults, then placed on media with RU486 for 7 days to induce transgene production. On day 7, they were switched to media without RU486 to halt Ub^6^-Stop expression and collected at different points. As shown in Fig. 4A, Ub^6^-Stop protein is mostly degraded within seven days, although we still observe it at 14 days. Thus, this protein is turned over in the intact fly. Next, we examined whether the proteasome regulates its levels.

We used RNA-interference (RNAi) to specifically knock down genes that encode proteasome components. Where available, we employed more than one RNAi line for each gene. We conducted RNAi-based studies in fly eyes, because knockdown of most of the genes we targeted is lethal when performed everywhere. We targeted α and β subunits of the 20S proteolytic core and the following components of the 19S proteasome: Rpn1 (helps with Ub binding and processing at the proteasome)^48^, Rpn9 (anchors other components to the 19S)^49^, Rpn11 (removes Ub chains)^1,25^, Rpt5 (facilitates interaction of 19S with 20S)^50^ and Rpn13 (Ub receptor)^51^. Targeting each of these subunits through RNAi led to higher levels of Ub^6^-Stop protein compared to controls; controls consisted of the background genetic line of RNAi constructs in the absence of any knockdown (Fig. 4B–E). Similarly, knockdown of Sem1 led to prominently higher levels of Ub^6^-Stop (Fig. 4D,E). Sem1 is necessary for 19S assembly^52^ and is a stoichiometric component of the 19S, where it functions as a Ub receptor^52–54^. Collectively, these results strongly implicate the proteasome in Ub^6^-Stop degradation.

Ubiquitinated proteins can come into direct contact with the proteasome, or can be assisted by Ub-binding proteins, referred to as proteasome shuttles. To examine the involvement of proteasome shuttles in Ub^6^-Stop turnover, we investigated the segregase, VCP, which functions in part to help deliver ubiquitinated proteins to the proteasome for degradation, as well as its cofactors, p47 and Ufd1-like^55–60^. We also targeted the proteasome shuttle protein Rad23^61–64^. Knockdown of VCP (Fig. 4C,E), p47 and Ufd1-like (Fig. 4D,E) each led to consistently higher protein levels of Ub^6^-Stop. Knockdown of Rad23 did not have as prominent of an effect on Ub^6^-Stop protein levels compared to p47, Ufd1-like and VCP (Fig. 4D,E).

**Figure 5:**
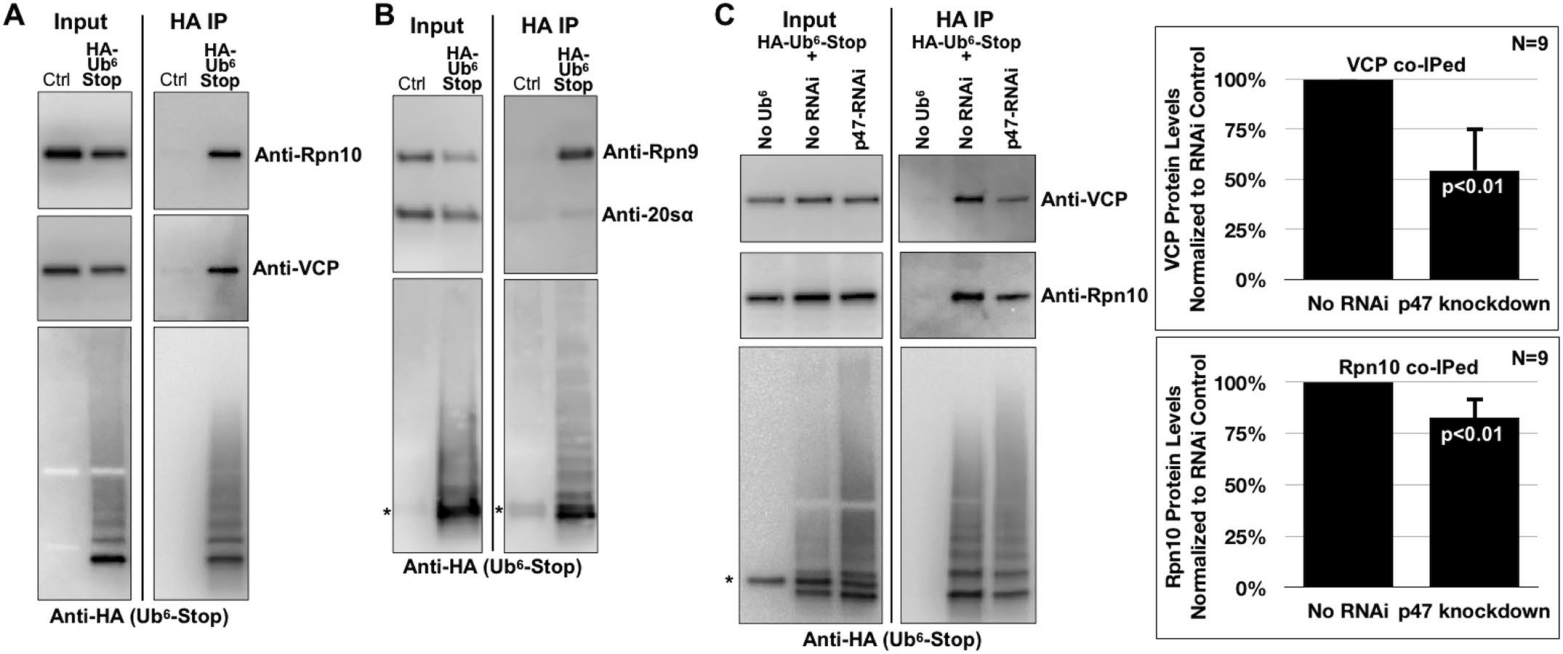
Ub^6^-Stop interacts with the proteasome, VCP and p47 in *Drosophila*. (**A–C**) Western blots of co-IPs from whole fly heads expressing Ub^6^-Stop in fly eyes. Flies were heterozygous for GMR-Gal4 and HA-tagged Ub^6^-Stop. Asterisks: non-specific bands in lanes not expressing HA-Ub^6^-Stop. Results are representative of experiments conducted independently at least three times, with similar results. In each panel, “Ctrl” signifies anti-HA-bead-bound antibody that was incubated with fly lysates that do not express any HA-tagged Ub^6^-Stop. For quantified blots in panel (C), we loaded gels to achieve comparable levels of Ub^6^-Stop in the IP lanes. The entire HA-Ub^6^ smear was quantified. Shown in histograms are means −/+ SD. P values are from two-tailed Student’s t-tests.

Subsequently, we examined the interaction of Ub^6^-Stop with VCP and the proteasome. We expressed Ub^6^-Stop in fly eyes, precipitated it under mild conditions and examined its interaction with endogenous Rpn9 (anchors Rpn10 to the 19S), Rpn10 (Ub receptor), 20Sα (proteolytic portion) and VCP^49,65,66^. Ub^6^-Stop co-precipitated VCP, Rpn10 and Rpn9. Unlike with the 19S components, we were unable to specifically co-precipitate 20Sα with Ub^6^-Stop (Fig. 5A,B). This could be due to dissociation of the 19S and 20S components during the IP. The rest of the results from panels 5A and B, however, indicate that Ub^6^-Stop comes into physical contact with the proteasome and VCP.

Lastly, we examined the effect of p47 in the interaction of Ub^6^-Stop with VCP and, downstream from this AAA ATPase, the proteasome^67^. We targeted this VCP cofactor based on data that it binds branched chains^8^ and because its knockdown has a clear impact on Ub^6^-Stop levels (Fig. 4). As shown in Fig. 5C, knocking down p47 leads to decreased levels of VCP and Rpn10 that co-precipitate with Ub^6^-Stop. Knockdown of p47 did not reduce the amount of Rpn10 that co-precipitates with Ub^6^-Stop as much as the levels of VCP that co-precipitates with Ub^6^-Stop. This may be due to VCP-independent routes through which Ub^6^-Stop reaches the proteasome. Collectively, data in Fig. 5 suggest that Ub^6^-Stop comes into contact with the proteasome at least in part through p47 and VCP.

### Ub^6^-GG can be conjugated *en bloc* in mammalian cells and in *Drosophila*

Our work thus far focused on Ub^6^-Stop, which cannot be conjugated onto other proteins. We studied these chains to examine the regulation of unanchored species when they cannot be cleaved into mono-Ub. Still, the prospect exists that unanchored chains, which normally contain a terminal “GG”, might become conjugated onto other proteins, effectively eliminating them from the unanchored pool. The possibility of *en bloc* conjugation of an entire Ub chain has been considered for some time in the Ub community^68–72^. We reasoned that our non-cleavable Ub species could be used to assess *en bloc* transfer within the frame of unanchored poly-Ub regulation.

**Figure 6:**
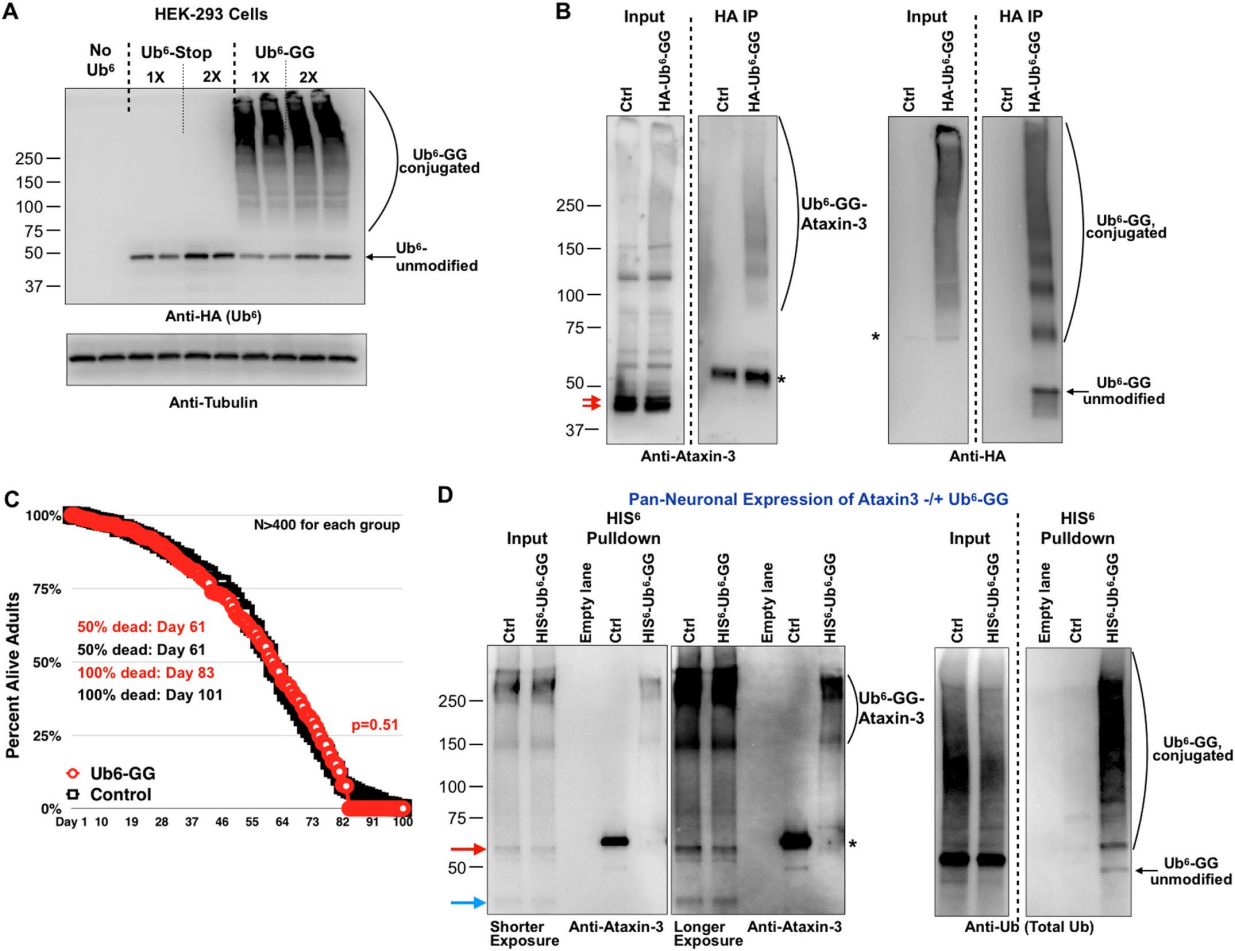
Ub^6^-GG is conjugated to proteins in mammalian cells and in flies. (**A**) HEK-293 cells were transiently transfected as indicated and harvested for whole lysate western blotting 24 hours later. No Ub^6^: empty vector control transfection. 1 × and 2 × denote relative transfection amounts. (**B**) HEK-293 cells were transfected with the empty host vector of Ub^6^-GG or with HA-tagged Ub^6^-GG for 24 hours. Cells were pelleted, lysed in RIPA buffer with protease inhibitors (PI) and subjected to a denature/renature protocol (Methods) before IP. Asterisks: non-specific signal. Red arrows: unmodified, full length ataxin-3 protein. Ataxin-3 runs as a doublet in input blots. This reflects the two alleles of the gene that have different CAG repeats. Dotted lines separate lanes from the same membrane, cropped and reorganized for ease of viewing. Un-organized blots are in Supplemental Figures. (**C**) Longevity from adult flies expressing, or not, Ub^6^-GG in all tissues via sqh-Gal4. P value was calculated using log-rank (Mantel-Cox) test. (**D**) Western blots from denature/renature HIS^6^-based precipitation of Ub^6^-GG expressed in all neurons alongside ataxin-3. Ctrl: no HIS^6^-Ub^6^-GG expressed alongside ataxin-3. Membrane was loaded with the same samples twice, cut in half and probed simultaneously with the indicated antibodies. We used a pan-Ub antibody in this case to show the specificity of the pulldown. Asterisk: non-specific band in the absence of HIS^6^-Ub^6^-GG expression in the fly. Red arrow: unmodified ataxin-3 protein. Blue arrow: ataxin-3 protein fragment most likely resulting from proteolytic cleavage^77^; this species of ataxin-3 is absent in IP lanes. Two different exposures are shown for anti-ataxin-3 signal. Dotted line separates lanes from the same membrane, cropped and reorganized for ease of viewing. Un-organized blots are in Supplemental Figures. For all panels, results are representative of experiments conducted independently at least three times, with similar results.

We transiently expressed Ub^6^-Stop and Ub^6^-GG in mammalian cells. As shown in Fig. 6A, expression of Ub^6^-GG leads to a smear above the unmodified band. Unlike in flies, we do not observe a smear above Ub^6^-Stop when it is transiently transfected in cells, even though it is degraded via the proteasome (Supplemental Fig. 2). Using stringent, denature/renature IPs of Ub^6^-GG from mammalian cells, we found an endogenous protein chemically modified by this chain, ataxin-3 (Fig. 6B), whose ubiquitination we have documented before^73–75^. Ataxin-3 is a DUB whose catalytic activity does not eliminate its own ubiquitination^76^. Mutations in the polyglutamine region of this DUB cause the neurodegenerative disease, Spinocerebellar Ataxia Type 3^77^. Unmodified ataxin-3 migrates at ~42 kDa. Ub^6^-GG migrates immediately below 50 kDa. We would expect ubiquitinated forms of ataxin-3 by Ub^6^-GG to appear ≥90 kDa. In Fig. 6B, the ataxin-3-positive smear from Ub^6^-GG denature/renature IPs begins below 100  kDa and extends all the way to the top, consistent with Ub^6^-GG-ataxin-3.

We then turned to flies. Expression of the non-cleavable, conjugatable Ub^6^-GG in all tissues does not cause marked lethality (Fig. 6C). To examine whether Ub^6^-GG can be utilized *en bloc* in flies, we again investigated ataxin-3, which is ubiquitinated in *Drosophila*^75,78^. Here, we used ataxin-3 with an expanded polyglutamine tract (from wild-type with ~20 repeats to pathogenic -range 77 glutamine residues), because this version of the protein is well ubiquitinated in the fly (our unpublished observations). We expressed ataxin-3 in the absence or presence of Ub^6^-GG pan-neuronally and utilized whole, intact flies to isolate HIS^6^-Ub^6^-GG under stringent, denaturing conditions. As shown in Fig. 6D, in the presence of Ub^6^-GG we observe ataxin-3 species in the high molecular weight portion of the pulldown lane, but not in the negative control lane, which has ataxin-3 but lacks Ub^6^-GG. We do not notice unmodified ataxin-3 species, intact or proteolytically cleaved, in either of the IP lanes (Fig. 6D). Polyglutamine-expanded, unmodified ataxin-3 migrates at ~60 kDa. Accounting for Ub^6^-GG (~50 kDa), we would expect the ubiquitinated species of ataxin-3 above the 100 kDa band, which is what we see (Fig. 6D). There isn’t a marked change in the higher molecular weight species of ataxin-3 in input lanes when Ub^6^-GG is expressed alongside this protein, but we clearly observe ataxin-3 signal in the Ub^6^-GG pulldown lane. Our interpretation of these results is that a portion of ataxin-3, in the high molecular weight part of the gel, is modified with Ub^6^-GG. Thus, some of ataxin-3 in the fly can be modified with the non-cleavable, conjugatable Ub^6^ species, without the need for this type of chain to be first cleaved into mono-Ub. We conclude that unanchored poly-Ub can be utilized *en bloc* for conjugation onto other proteins in an intact organism, without first being disassembled into mono-Ub.

## Discussion

**Figure 7:**
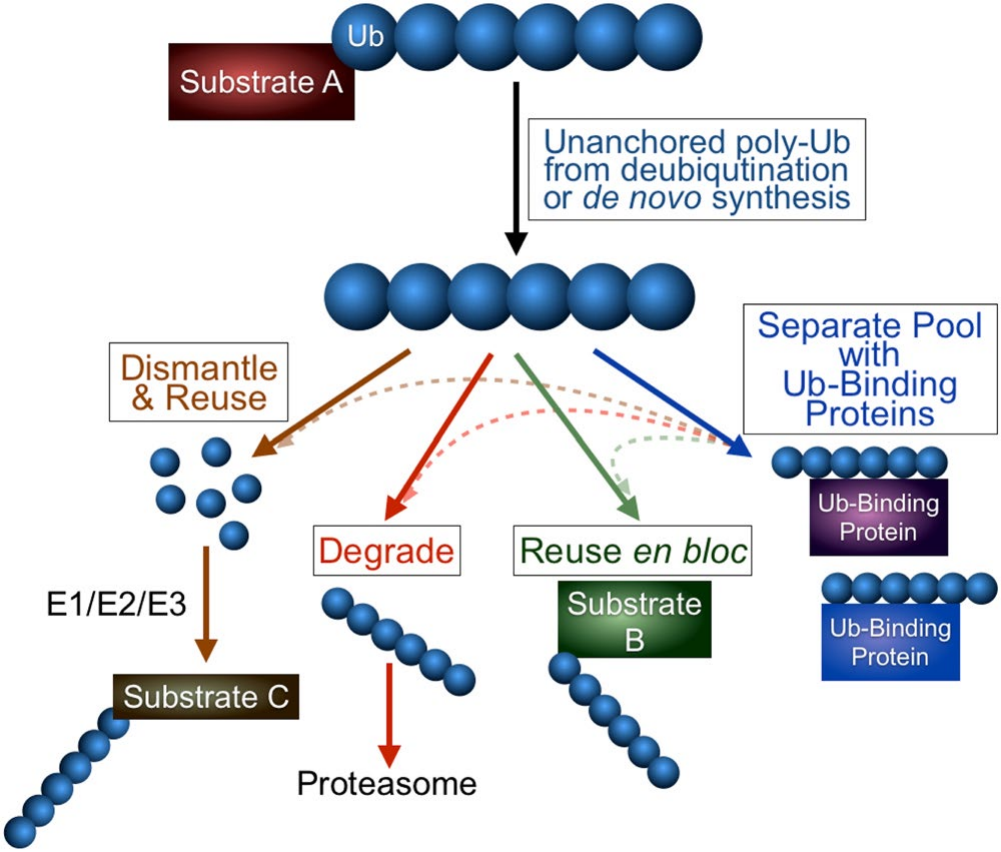
Model of unanchored poly-Ub regulation *in vivo*. Unanchored poly-Ub can be dismantled into mono-Ub and reutilized in future Ub reactions by the coordinated action of E1/E2/E3. This is the canonical model of Ub recycling. Our results in *Drosophila* suggest the possibility of other, non-mutually exclusive mechanisms of unanchored Ub chain control: their degradation as whole units by the proteasome; their reutilization *en bloc*, enabling the conjugation of the entire chain onto a substrate without the prior need for dismantling into mono-Ub; or their maintenance in a separate, “reserve” pool through interaction with Ub-binding proteins. The “reserve” pool could feed into the other routes.

We set out to examine what happens to unanchored chains when they cannot be disassembled by DUBs. We generated new *Drosophila* models of poly-Ub, which we believe will find future use in the fly and ubiquitin communities. The unanchored chains that we constructed exist in different lengths and topologies *in vivo*, from unmodified Ub^6^ to markedly higher molecular weight species, and contain various linkages. Nearly 75% of Ub^6^ appears as modified bands/smears conjugated with endogenous Ub. These free chains were degraded by the proteasome and could also be attached onto other proteins, without the need to be first deconstructed into mono-Ub. Based on these findings, we propose a model of unanchored chain management that comprises four potential routes (Fig. 7): the first is canonical, where unanchored poly-Ub is dismantled and then reutilized^2,5,17,22,25^. Besides it, we posit that there may be additional options for unanchored chains: they can be degraded by the proteasome, they can be eliminated by being used *en bloc* to be conjugated onto other proteins in the cell, or they can be bound by Ub-binding proteins and maintained in a separate or “reserve” pool until they re-enter utilization, if or when needed. The last possibility is not necessarily mutually exclusive with the other potential avenues and may serve as a feeding route for the other options. How is the decision made to degrade or reutilize an unanchored chain rather than dismantle it? Under some conditions, it might be more advantageous to use a ready-made chain than to make a new one to attach to a specific substrate, perhaps during low energy states or when increased rates of protein turnover are required—in other words, chain shuffling among different proteasome substrates could enhance their targeting and degradation.

The unanchored chains that we designed and expressed in flies do not represent all of the various types of Ub linkages and species that can be found in the cell. In fact, linear chains are a minor constituent of the total Ub pool^79^ and the functions of these types of chains are not entirely known^80^, hindering to some extent the utility of the lines that we generated toward understanding more broadly Ub-dependent pathways. Still, the linear Ub^6^ that we constructed exist as modified and unmodified species of various lengths and topologies in *Drosophila*. We contend that these species represent different types of free poly-Ub *in vivo* and, at the very least, can be used to provide clues into regulatory mechanisms that dictate what happens to branched chains containing multiple Ub-Ub linkages. It was recently reported that branched chains consisting of K48 and K11 linkages are generated by E2/E3 complexes and play critical roles in targeting misfolded proteins for proteasomal degradation^8^.

The VCP cofactors Ufd1-like and p47 as well as VCP itself are critical for the levels of unanchored poly-Ub. The proteasome-associated protein, Rad23 also appeared important in this process. We did notice variation in the extent of effect with different RNAi lines targeting a specific gene; this is most likely due to differences in the efficacy of each line in reducing the mRNA levels of the targeted fly gene. Based on our results, VCP and its co-factors are key players for the protein levels of unanchored poly-Ub in the fly. This is not surprising for chains comprising various linkages (K48, K63 and K27, at least), since p47 and VCP are adept at recognizing branched chains in mammalian cells^8^.

Unanchored chains have been argued to compete with ubiquitinated proteasome substrates for access to the 26S proteasome^23,25,81,82^. However, unanchored poly-Ub species that we induced in the fly do not negatively impact degradation of proteasome substrates, even though these chains interact with the proteasome. We examined two substrates, endogenous cyclin A and the reporter CL1-GFP. The protein levels of neither substrate were increased. In fact, we observed lower protein levels of CL1-GFP, indicative of enhanced degradation of this fusion protein. We also did not observe changes in the levels of VCP, which is upregulated when the fly proteasome is inhibited^41^. Our results suggest that unanchored chains need not play an exclusively inhibitory role for the proteasome. Binding of free poly-Ub to various Ub-binding proteins could sequester the chains away from the proteasome, keeping the degradative machinery unperturbed. But, what might account for lower levels of CL1-GFP? For a subset of proteins degraded by the proteasome, unanchored poly-Ub might enhance substrate delivery to this machinery as a result of recruitment of Ub-binding proteins that normally delay their degradation, but which are now occupied with free poly-Ub. In the case of CL1-GFP, it might be that a protein that would normally bind to CL1-GFP and restrain its degradation is now occupied with Ub^6^, leading to more prompt degradation of CL1-GFP. Other possibilities exist. Future work is required to untangle these and other details.

The discrepancy between our work and prior reports that unanchored chains impede proteasome activity^23,25,81,82^ could be due to various reasons and highlights a need for additional studies of unanchored poly-Ub regulation. Some earlier work was conducted in cultured mammalian cells using transient expression. Perhaps, unanchored poly-Ub transiently inhibits the proteasome and becomes toxic in isolated cells, whereas *in vivo* free chains are easily managed. Other work focused on the function of specific DUBs, such as USP5, whereas we assessed unanchored poly-Ub more directly. Proteasome inhibition when certain DUBs are absent might result from perturbation of specific substrates rather than general effects from unanchored poly-Ub. For example, similar to others^26^, we observed impeded proteasome function when USP5 was knocked down in the fruit fly^27^. However, USP5 mutation or knockdown might lead to inhibited proteasome activity as a result of accumulation of its substrates, independently of unanchored chains. In fact, not all proteasome substrates are impacted by USP5 knockdown^82^ and there is evidence that USP5 has specific substrates^29,83,84^. The tools that we generated here will be beneficial for future work to continue assessing the consequences of unanchored poly-Ub in intact organisms.

Lack of consistent and marked lethality from unanchored chains during development or in adults, including under heat stress, suggests that they are not necessarily toxic. Nonetheless, conditions or tissues where unanchored poly-Ub can be problematic may exist. For example, we did observe mild reduction in the lifespan of flies expressing Ub^6^-Stop in glial cells or neurons specifically. A study of ubiquitin homeostasis at the *Drosophila* neuromuscular junction (NMJ) during larval development^85^ utilized mono-Ub transgenes that can be modified with endogenous Ub, but which cannot themselves be added onto other substrates because they lack a terminal “GG”. Post-synaptic presence of these transgenes caused morphological anomalies under some circumstances, but not others. If K48 linkages could be attached onto the transgene, there were mild, but statistically significant, anomalies. If K48 linkages could not be made onto the mono-Ub transgene (other chains could be constructed) there was no toxicity^85^. Thus, some types of unanchored linkages appeared mildly problematic in this assay, while others did not. Whether NMJ anomalies disappeared with continued development, or if these constructs were lethal throughout the fly was not clear. Together with our results, this earlier study^85^ substantiates the conclusion that unanchored chains need not be toxic. Although we used robust drivers without devastating lethality effects, we will not discount the possibility of anomalies caused by free chains at very high levels, under certain stressors, or in specific types of assays. The point we want to highlight is that unanchored chains can be handled well *in vivo* and that the extent of their toxicity should be reevaluated.

In summary, unanchored chains can be managed *in vivo* in ways that do not require their disassembly by DUBs: they can be degraded and they may even be conjugated *en bloc* to other proteins. Our work presents new possibilities into Ub recycling and reutilization.

## Methods

### Antibodies

Anti-ataxin-3 (1:15000; MJD, rabbit polyclonal, ref.^86^); anti-HA (1:1000; rabbit monoclonal; Cell Signaling Technology, #3724); anti-Ub (1:500; rabbit polyclonal; DAKO, #Z0458); anti-Tubulin (1:10000, mouse monoclonal, Sigma-Aldrich, #T5168); anti-Rpn10 (1:2000; rabbit polyclonal; AbCam, #ab18512); anti-20Sα (1:100; mouse monoclonal, Santa Cruz Biotech, #sc-65755); anti-Rpn9 (1:100; mouse monoclonal, Santa Cruz Biotech, #sc-65754); anti-Rpt6 (1:1000; rabbit polyclonal; Cell Signaling Technology, #13392); anti-VCP (1:1000; rabbit polyclonal; LSBio, #LS-C313248); anti-K63 (1:1000; rabbit monoclonal; Cell Signaling Technology, #5621); anti-K48 (1:1000; rabbit monoclonal; Cell Signaling Technology, #8081); anti-K27 (1:2000; rabbit polyclonal, Advanced Biomart, #FPA-21344M); anti-CycA (1:200; mouse monoclonal, Developmental Studies Hybridoma Bank at the University of Iowa, #A12); anti-actin (1:200; mouse monoclonal, Developmental Studies Hybridoma Bank at the University of Iowa, #1E12); anti-Lamin (1:200; mouse monoclonal; Developmental Studies Hybridoma Bank at the University of Iowa, ADL84.12); anti-GFP (1:1000; mouse monoclonal, Millipore, #MAB3580); goat anti-mouse, peroxidase conjugated secondary (1:5000; Jackson Immunoresearch); goat anti-rabbit, peroxidase conjugated secondary (1:5000; Jackson Immunoresearch). Antibodies against K27, K48 and K63 linkages were the only antibodies we were able to obtain.

### Construct generation

Ub^6^ transgenes (Fig. 1A) were synthesized by GenScript and cloned into pWalium10.moe. Purified plasmid was injected into yw; attP40 by Duke University Model Systems. The transformed chromosome was migrated onto w^1118^ parental line. The Ub transgenes were subcloned into pcDNA3.1-HA vector for mammalian expression and into pGEX-6P1 for recombinant production in bacteria.

### *Drosophila*-related procedures and stocks

In all panels and figures, flies were heterozygous for driver and transgenes. Flies were maintained in diurnal incubators at 25 °C and ~60% humidity, in conventional cornmeal media. Where noted, RU486 was used in the same media, as previously described^87^. Where noted, adults were maintained at 30 °C and ~60% humidity. Tubulin-Gal4-GS was a generous gift of Dr. R. J. Wessells, Wayne State University; sqh-Gal4 was originally gifted by Dr. Daniel Kiehart, Duke University; Mef2-Gal4 (#27390), elav-Gal4 (#458) and GMR-Gal4 (#8121) were from Bloomington *Drosophila* Stock Center; repo-Gal4 was gifted by Dr. Daniel Eberl, University of Iowa. The ataxin-3 lines have been described before^45–47^. The following RNAi lines were from the Bloomington *Drosophila* Stock Center: VCP (#32869, #35608), Rad23 (#44031, #44465), Rpt5 (#53886), Rpn1 (#34348), Ufd1-like (#41823), prosalpha2 (#36898), prosalpha3 (#55217), prosbeta5 (#34810), Rpn9 (#34034). The following RNAi lines were from the Vienna *Drosophila* RNAi Center: Rad23 (#30498), Rpn11 (#19272), Sem1 (#31787, #31789, #49152, #49153), Ufd1-like (#24700, #10473), p47 (#17529, #10748). For fly longevity, male and female adults were collected after eclosion and aged in conventional cornmeal fly media at 25 °C, unless otherwise noted. The total number of flies per vial was ~20. Flies were transferred to new vials every 2–3 days, until all were dead.

### Western Blotting

Five whole adults flies, or ten dissected fly heads per group were homogenized in hot lysis buffer (50 mM Tris pH 6.8, 2% SDS, 10% glycerol, 100 mM dithiothreitol), sonicated, boiled for 10 min, and centrifuged at top speed at room temperature for 10 min. Western blots were developed and quantified using a CCD-equipped VersaDoc 5000MP system and Quantity One software (Bio-Rad), as described previously^73,88,89^. For transfected cells, media was removed, cells were rinsed with ice-cold PBS and lysed in hot lysis buffer, boiled for 10 minutes and spun for 10 minutes at max speed at RT.

### Immunoprecipitation & subcellular fractionation

For stringent precipitation of HA-Ub^6^ from flies, 30 flies per group were homogenized in RIPA lysis buffer (50 mM Tris, 150 mM NaCl, 0.1% SDS, 0.5% deoxycholic acid, 1% NP40, pH 7.4) supplemented with complete protease inhibitor cocktail (PI; Sigma-Aldrich), sonicated, centrifuged at 15000 × g for 20 minutes at 4 °C. Supernatant was denatured for 30 min with 1% final SDS at RT, renatured for 30 min with final 4.5% TritonX-100 at RT, and then incubated with anti-HA antibody-bound beads (Sigma-Aldrich) for 4 hours tumbling at 4 °C. Beads were rinsed 5 × with RIPA, twice at 4 °C for 5 minutes, and bead-bound complexes were eluted with SDS loading buffer and boiling for 5 minutes. For co-IPs under gentler conditions from the same flies, NETN lysis buffer (50 mM Tris pH 7.5, 150 mM NaCl, and 0.5% IPEGAL ca-630) was used instead, and the supernatant was incubated with beads without a denature/renature step, rinsed 4 × with NETN and bead-bound complexes were eluted with SDS loading buffer and heat. Precipitations for HIS^6^-tagged Ub^6^ were conducted differently. Flies were homogenized in Buffer 1 (50 mM Tris pH 8, 6 M guanidine HCl, 10 mM imidazole) supplemented with PI, sonicated, centrifuged as above and the supernatant was incubated with Ni-NTA beads (Qiagen) for 2 hrs at 4 °C. Afterwards, beads were rinsed 6 × each with Buffer 1, Buffer 2 (50 mM Tris pH 8, 150 mM NaCl, 8 M urea, 20 mM imidazole), and Buffer 3 (50 mM Tris pH 8, 500 mM NaCl, 20 mM imidazole). Complexes were eluted with final 250 mM imidazole in Buffer 3. For stringent, HA-based purification from mammalian cells, pelleted cells (in ice-cold PBS) were lysed in RIPA buffer + PI, sonicated, centrifuged (15000 × g, 20 minutes, 4 °C) then denatured for 30 min at RT with 1% final SDS, renatured at RT with final 4.5 × TritonX-100 and incubated with anti-HA bead-bound antibody for 4 hours at 4 °C. Beads were then rinsed 10 × with RIPA + PI and protein was eluted with SDS loading buffer and heat. In all precipitation experiments, controls included bead-bound antibodies (for HIS^6^ pulldowns, Ni-NTA resin) with lysate lacking the protein targeted by the antibody/resin. For subcellular fractionation, 5 flies per group were used with the ReadyPrep Protein Extraction Kit (Cytoplasmic/Nuclear; Bio-Rad). Flies were lysed in cytoplasmic extraction buffer and nuclei were resuspended in protein solubilization buffer. Samples were analyzed by western blotting.

### Recombinant protein preparation and *in vitro* deubiquitination

Recombinant Ub^6^ was produced in bacteria using previously published protocols, and eluted from glutathione sepharose beads using PreScission Protease (GE Healthcare)^27,28,73,74,88,90,91^. For *in vitro* deubiquitination assays with recombinant Ub^6^ and USP5, we utilized methods previously described^27,73–75,88,92^. In brief, Ub^6^ and USP5 were produced in bacteria, and eluted from glutathione sepharose beads by PreScission Protease (GE Healthcare). 1 µM (final) Ub^6^ or 1 µM (final) commercial Ub chain (Boston Biochem) and 50 nM (final) USP5 were combined together in kinase reaction buffer (0.5 M Tris pH 7.5, 0.5 M KCl, 0.2% DTT) and incubated at 37 °C for the indicated amounts of time. Fractions were collected at the indicated time points and reactions were stopped by adding SDS loading buffer and boiling for 1 minute. For deubiquitination of ubiquitinated Ub^6^ species from *Drosophila* lysates, 40 whole flies expressing HA-Ub^6^-STOP in all tissues (sqh-Gal4 was the driver) were homogenized in RIPA lysis buffer + PI, sonicated, centrifuged (15 min, 15000 × g at 4 °C) and supernatant was incubated with anti-HA bead-bound antibody (Sigma-Aldrich) for 1.5 hr. Beads were rinsed 10X with RIPA + PI and 3X with kinase buffer, split equally and one side was supplemented with additional PI, whereas the other was supplemented with 100 nM (final) USP2 catalytic domain (Boston Biochem). Reactions were incubated at 37 °C for the times indicated in figures and reactions were stopped by the addition of 2X SDS sample buffer and by boiling for 1 minute.

### Mammalian cells and procedures

HEK-293 and HeLa cells were from ATCC and were grown in DMEM with 10% FBS and 5% Penicillin-Streptomycin under conventional conditions. Cells were transfected using Lipofectamine LTX (Invitrogen) as directed by the manufacturer. Twenty-four hours after transfection, cells were harvested in boiling SDS lysis buffer.

### Data availability

All pertinent data for this work are included. Independent repeats not included but referred to in text or numerically summarized in graphs and tables can be requested by contacting the corresponding author.

## Electronic supplementary material

Supplementary Information
