# Supplementary material for: Expression and Regulation of Deubiquitinase-Resistant, Unanchored Ubiquitin Chains in Drosophila
Source: Sci Rep. 2018 May 31;8:8513. doi: 10.1038/s41598-018-26364-x (PMC5981470; doi:10.1038/s41598-018-26364-x)
Supplement: Supplementary file 1 — Supplementary Information [file 41598_2018_26364_MOESM1_ESM.pdf]

Supplemental Figures For

Expression and Regulation of Deubiquitinase-Resistant, Unanchored Ubiquitin Chains in *Drosophila*

Jessica R. Blount, Kozeta Libohova, Gregory B. Marsh,
Joanna R. Sutton, Sokol V. Todi

Supplemental Figure 1

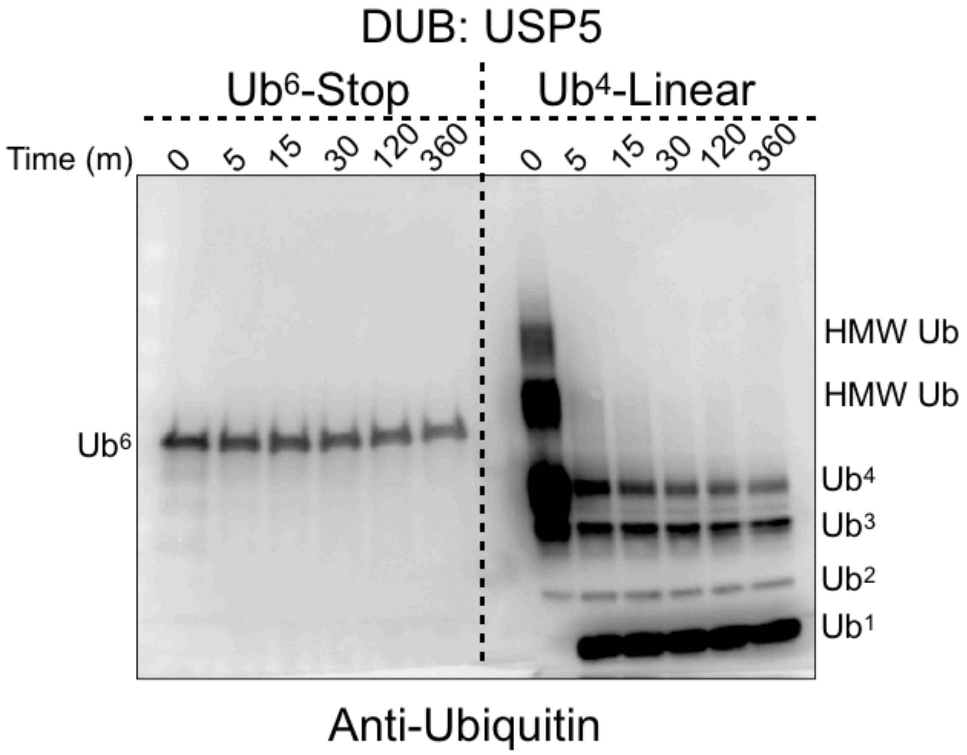

Supplemental Figure 1: USP5 does not dismantle recombinant Ub^6-Stop *in vitro*

Recombinant Ub^6-Stop (1μM) was generated in bacteria, cleaved from GST and incubated with USP5 (50 nM) for the indicated amounts of time. Reaction was stopped with SDS and heat. Results are representative of experiments conducted independently at least three times, with similar results. The lot of linear chains purchased for these reactions was different than the one in figure 1B. Some of the linear chains we procure from Boston Biochem show marked levels of higher molecular weight species (HMW), which are most likely longer species of the same linkage. Regardless, USP5 is able to quickly cleave those chains, but not Ub^6-Stop.

Supplemental Figure 2

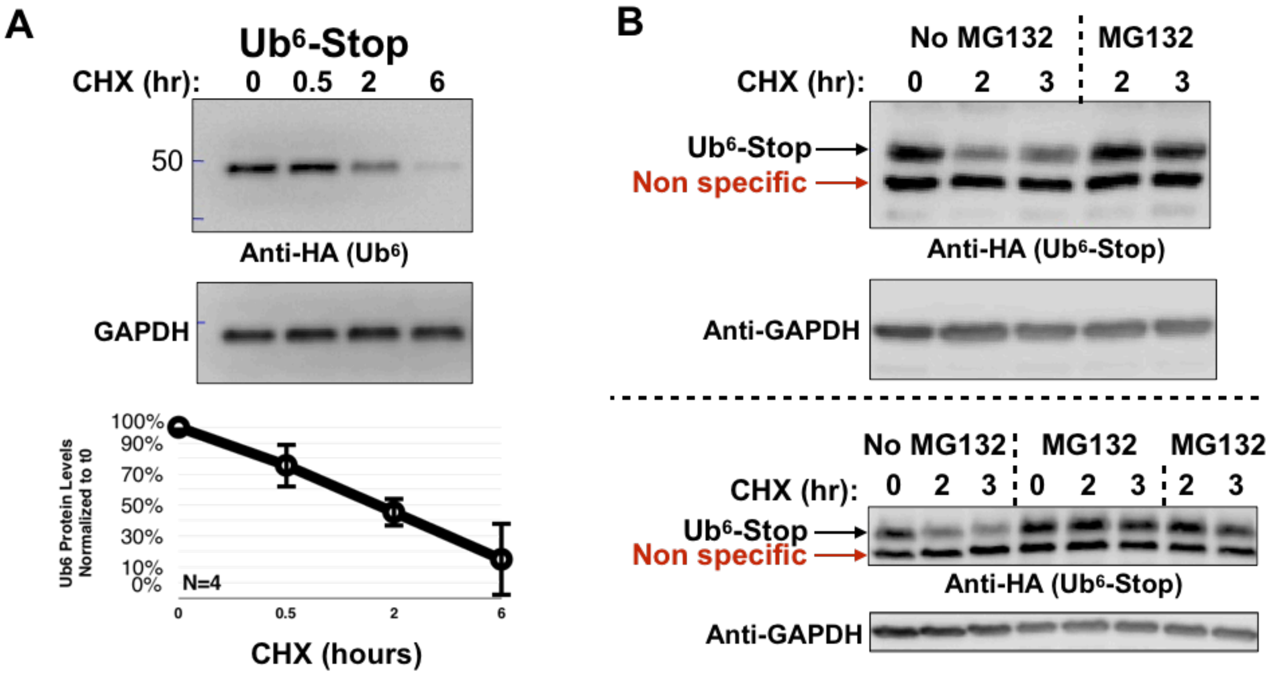

Supplemental Figure 2: Turnover of Ub6-Stop in mammalian cells.

A) HeLa cells were transiently transfected with HA-tagged Ub^6-Stop for 24 hrs using Lipofectamine LTX (Invitrogen), then cycloheximide (CHX; AG Scientific) was added at a final concentration of 100µg/ml for the indicated times to stop translation of new proteins. Blots are from whole cell lysates. Graph: quantification of signal from the blots above and other, independent experiments.

B) HeLa cells were transfected with Ub^6-Stop for 24 hours, then incubated, or not, with the proteasome inhibitor, MG132 (10µM final, Boston Biochem) as well as CHX, as indicated. Blots are representative of experiments conducted at least 4 independent times, with similar results. MG132 loads are from different experimental repeats.

Select
Un-cropped images
and different exposures

1D

Ubiquitous Expression

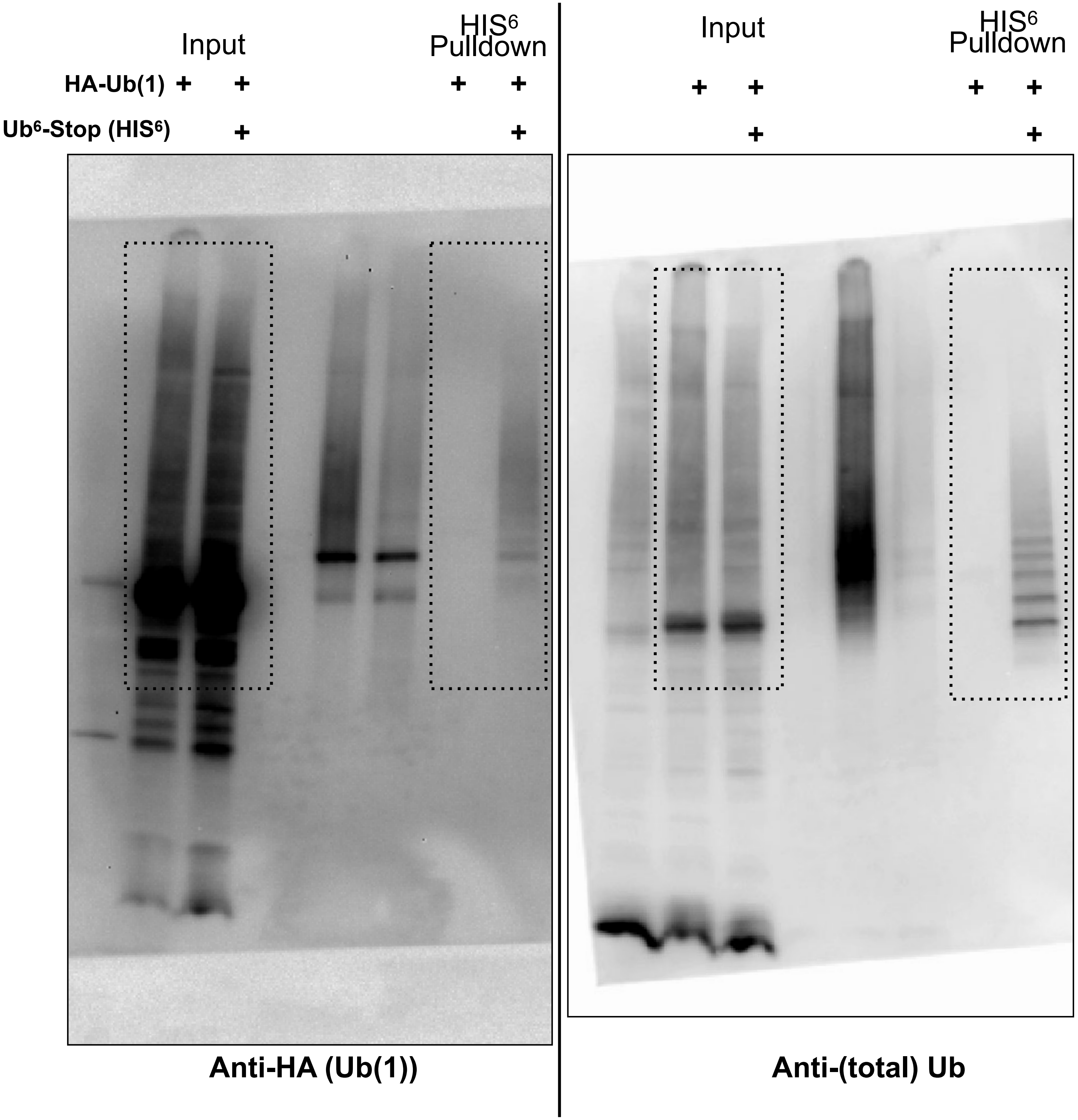

Boxes: areas in main figure

1G

All Ub6-Stop

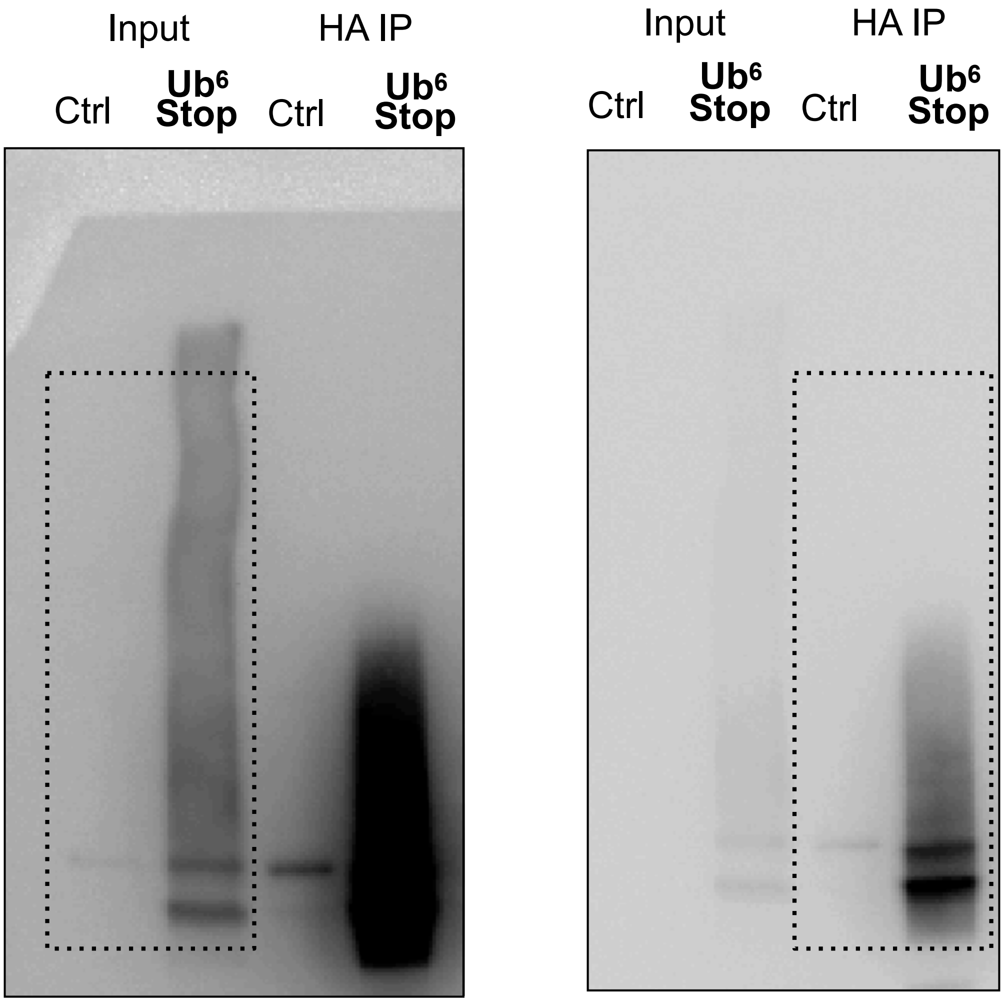

Anti-HA Ub6-Stop

Two different exposures are shown of the same membrane because input and IP lanes were chosen from these blots for the main figures. Exposures: Longer, shorter.

1G

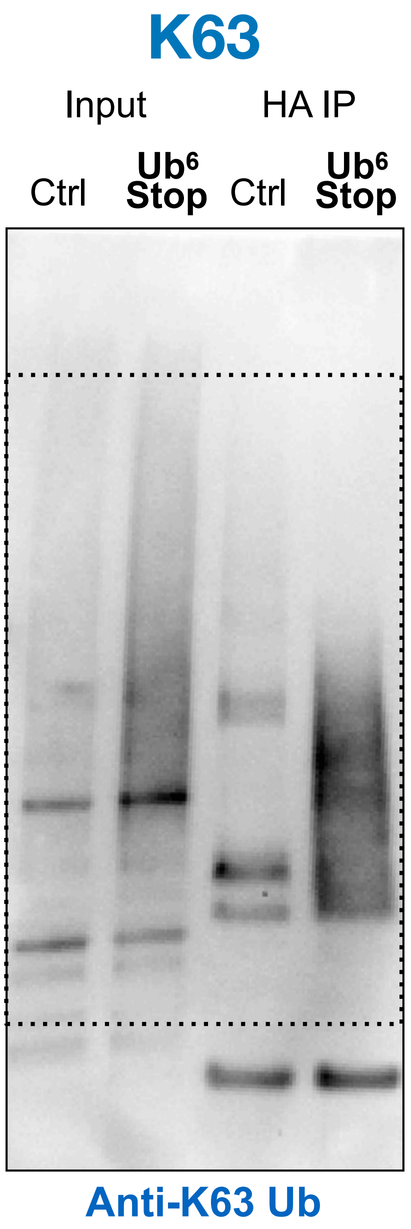

1G

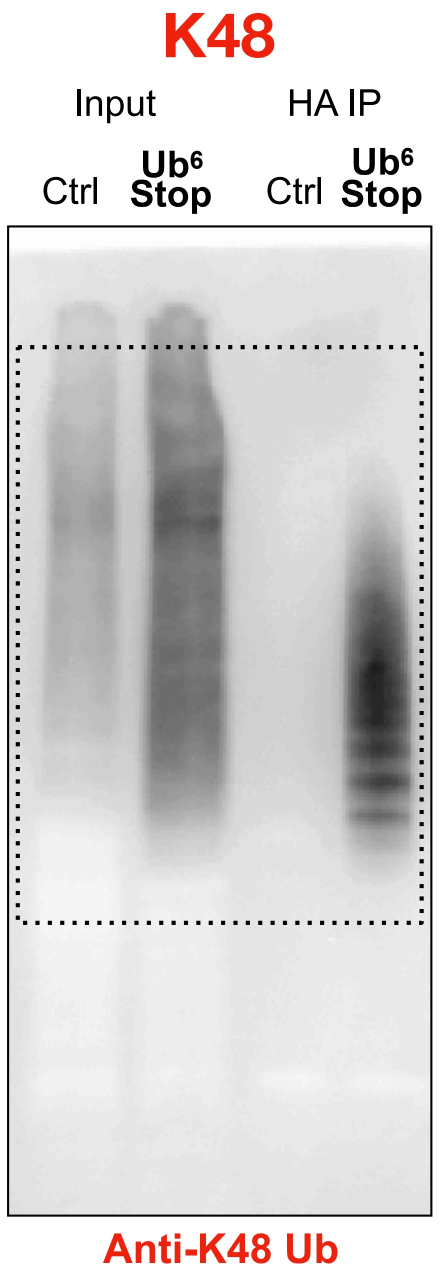

1G

K27

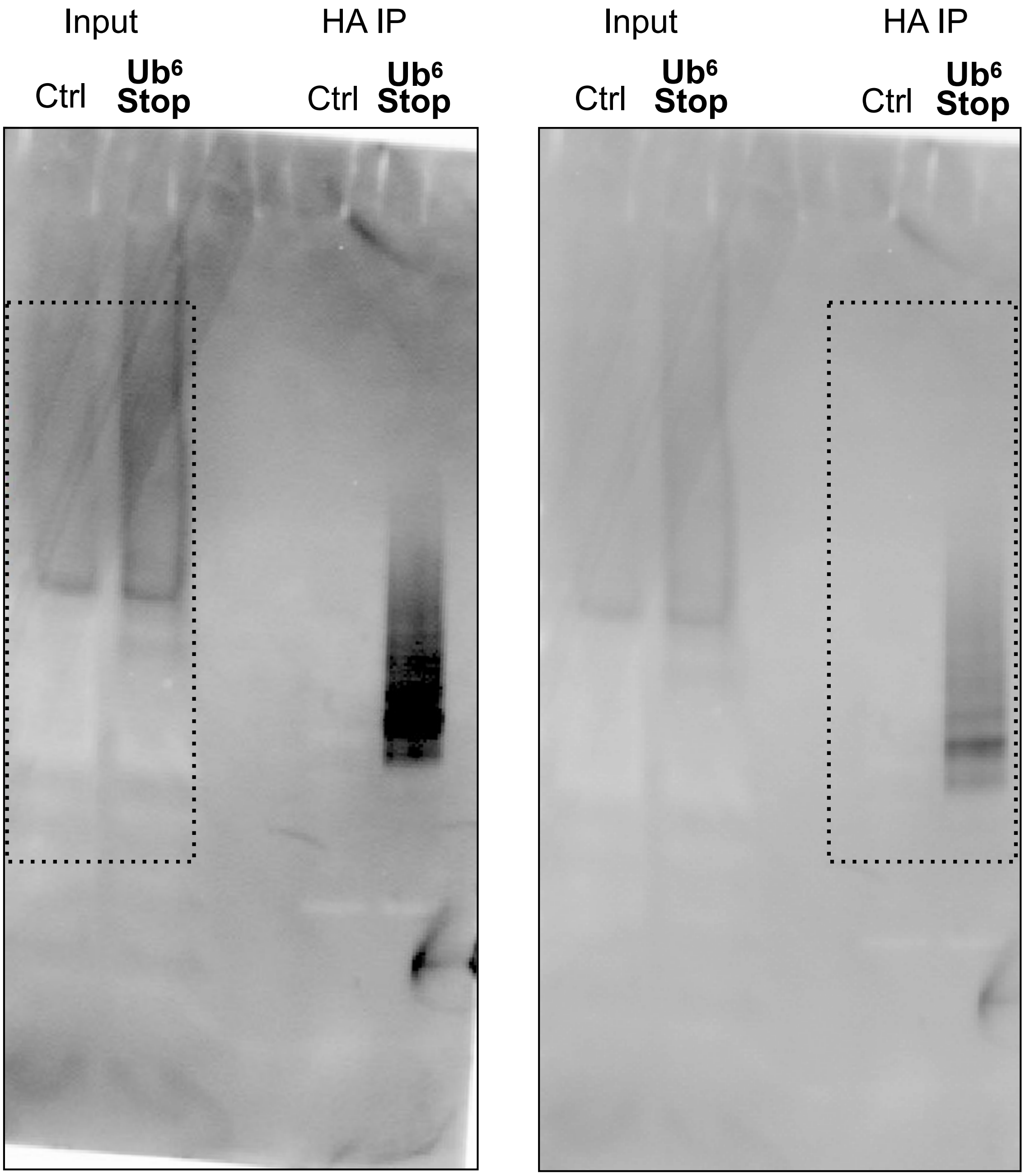

Anti-K27 Ub

Two different exposures are shown of the same membrane because input and IP lanes were chosen from these blots for the main figures. Exposures: Longer, shorter.

2B - membrane was probed with GFP and tubulin antibodies at the same time

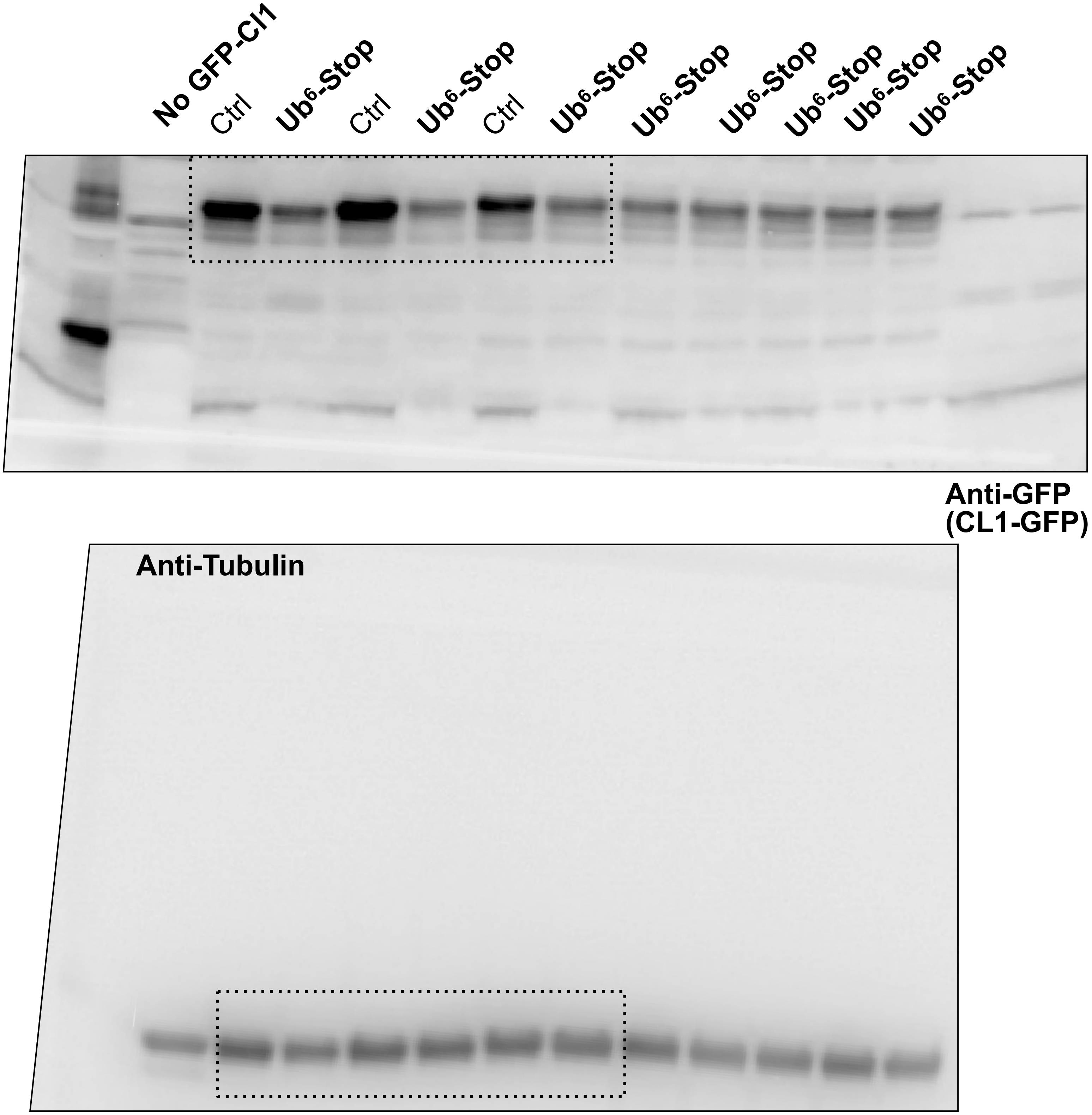

Boxes: areas in main figure

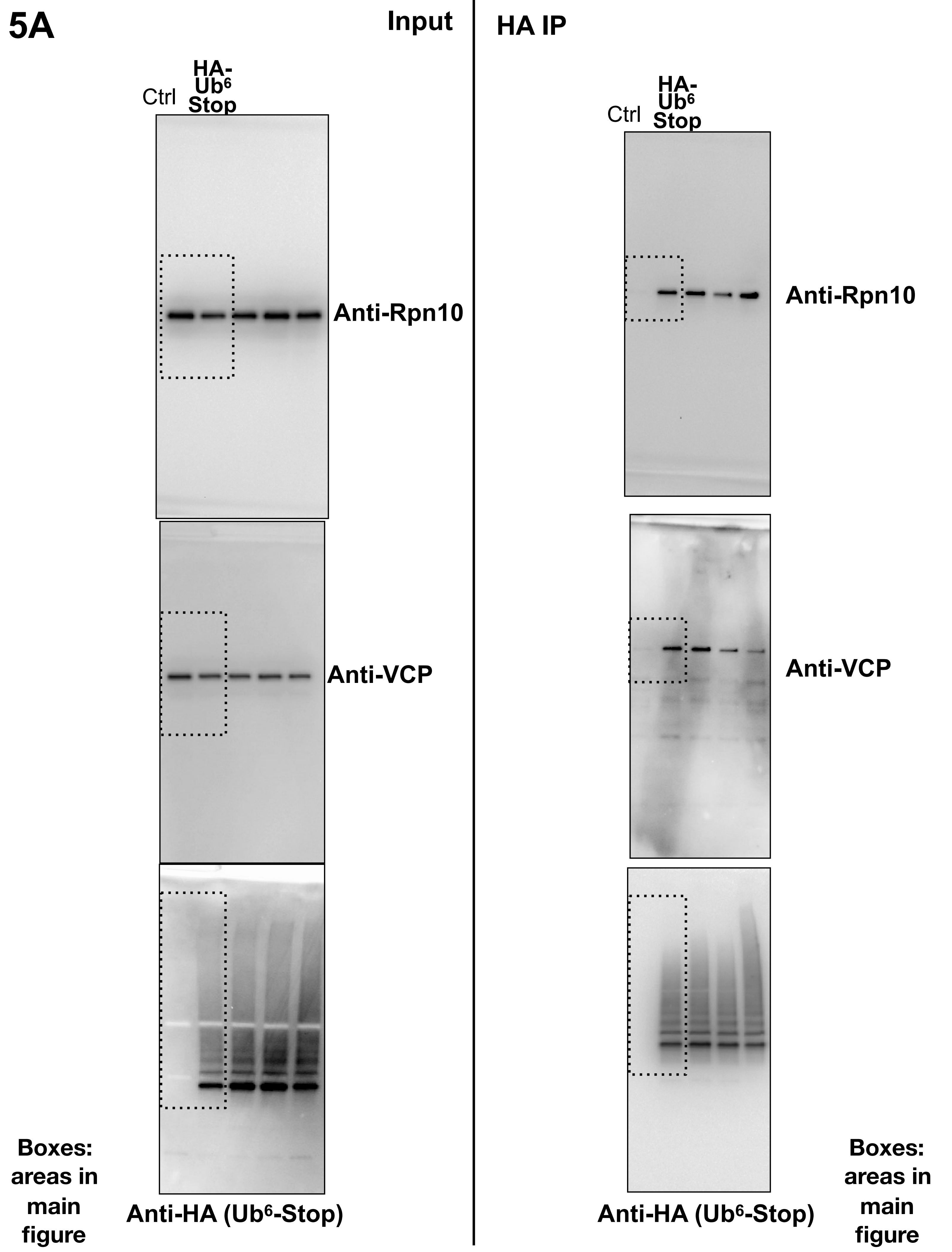
5A
Input
HA IP
Ctrl
HA-
Ub6
Stop
Anti-Rpn10
Anti-VCP
Anti-HA (Ub6-Stop)
Boxes: areas in main figure

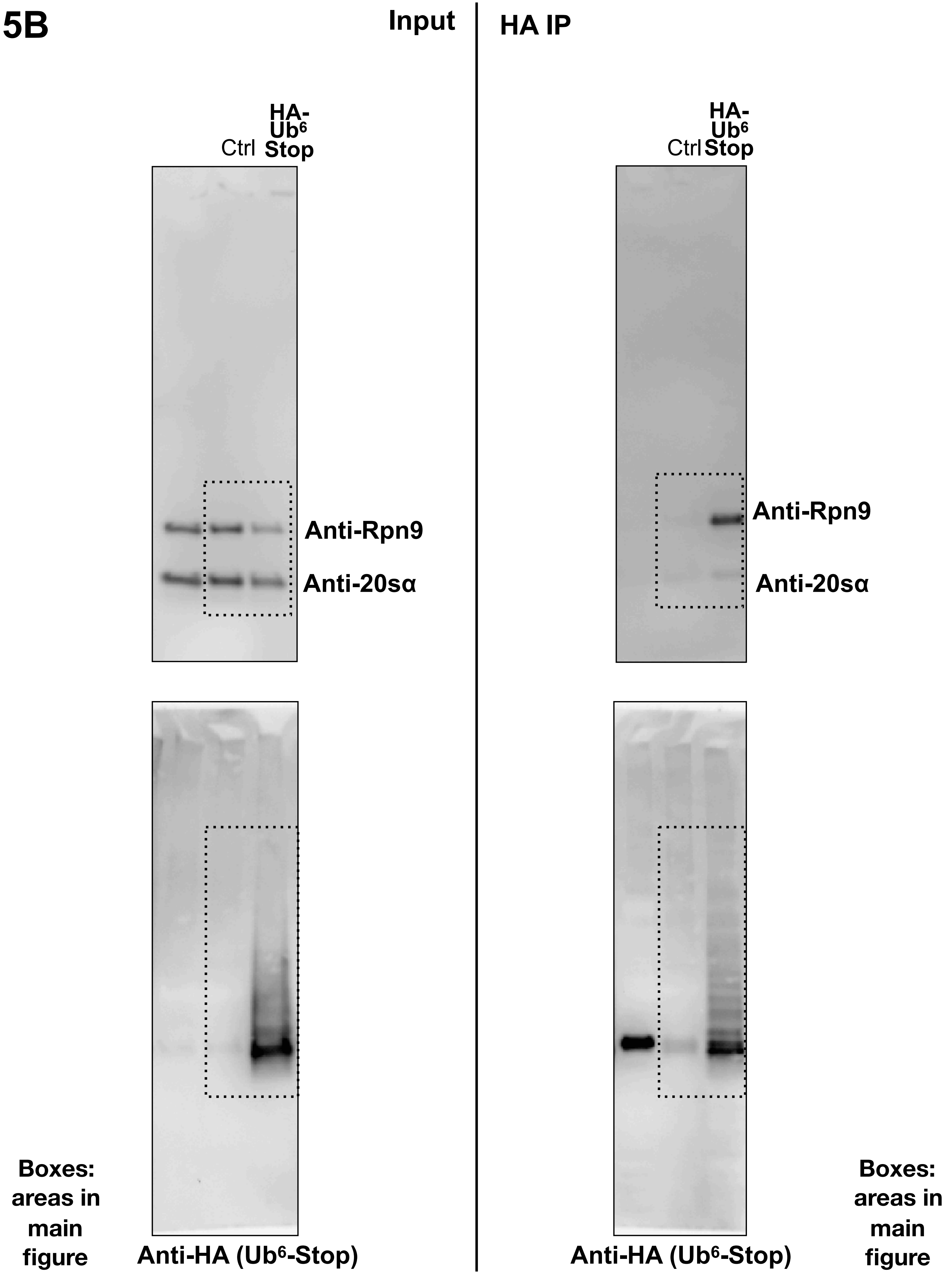
5B
Input
HA IP
Ctrl
HA-Ub6 Stop
Ctrl
HA-Ub6 Stop
Anti-Rpn9
Anti-20sα
Anti-Rpn9
Anti-20sα
Boxes: areas in main figure
Anti-HA (Ub6-Stop)
Anti-HA (Ub6-Stop)
Boxes: areas in main figure

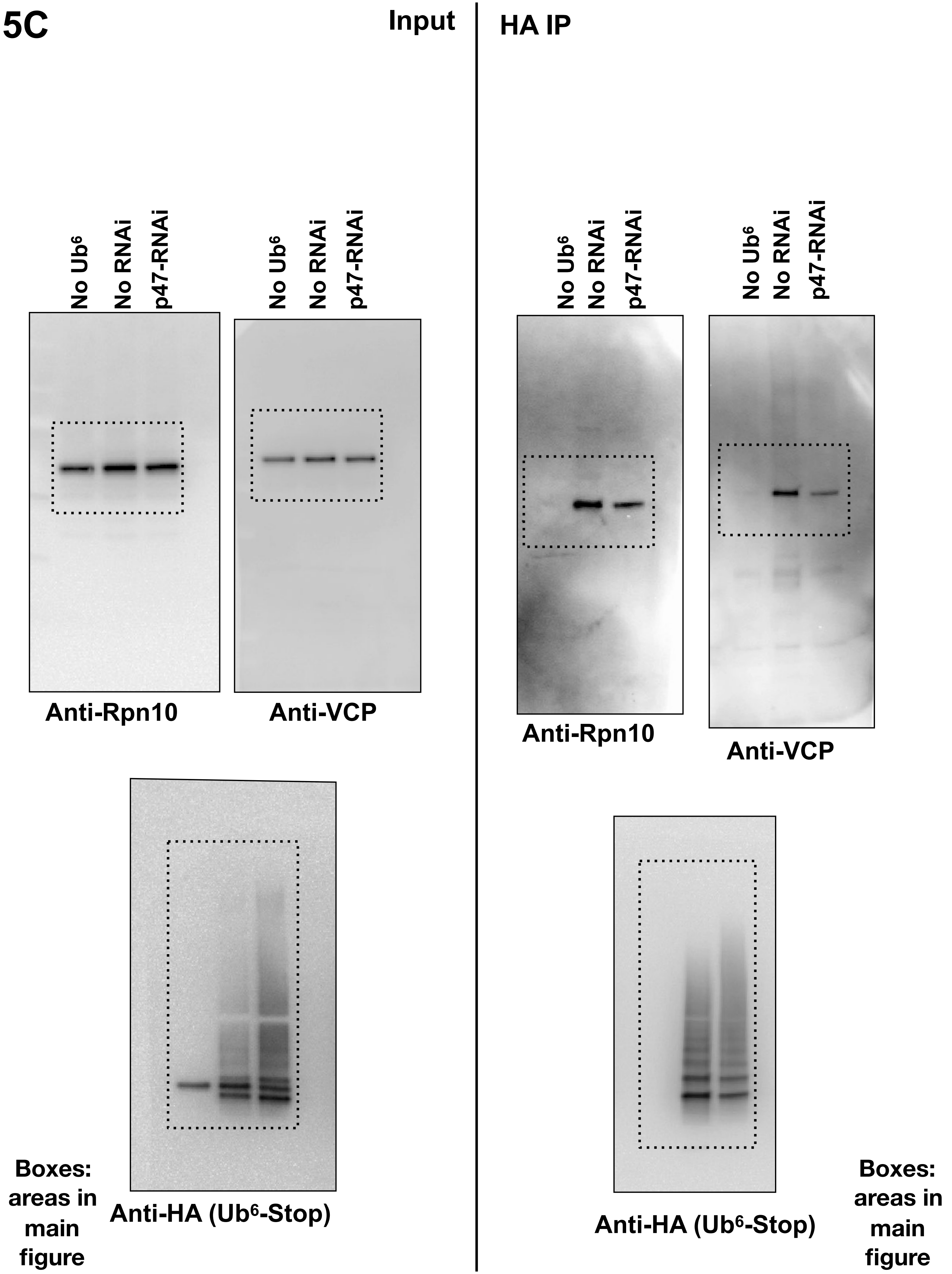

5C
Input
HA IP
No Ub6
No RNAi
p47-RNAi
No Ub6
No RNAi
p47-RNAi
Anti-Rpn10
Anti-VCP
No Ub6
No RNAi
p47-RNAi
No Ub6
No RNAi
p47-RNAi
Anti-Rpn10
Anti-VCP
Anti-HA (Ub6-Stop)
Anti-HA (Ub6-Stop)
Boxes: areas in main figure
Boxes: areas in main figure

6B

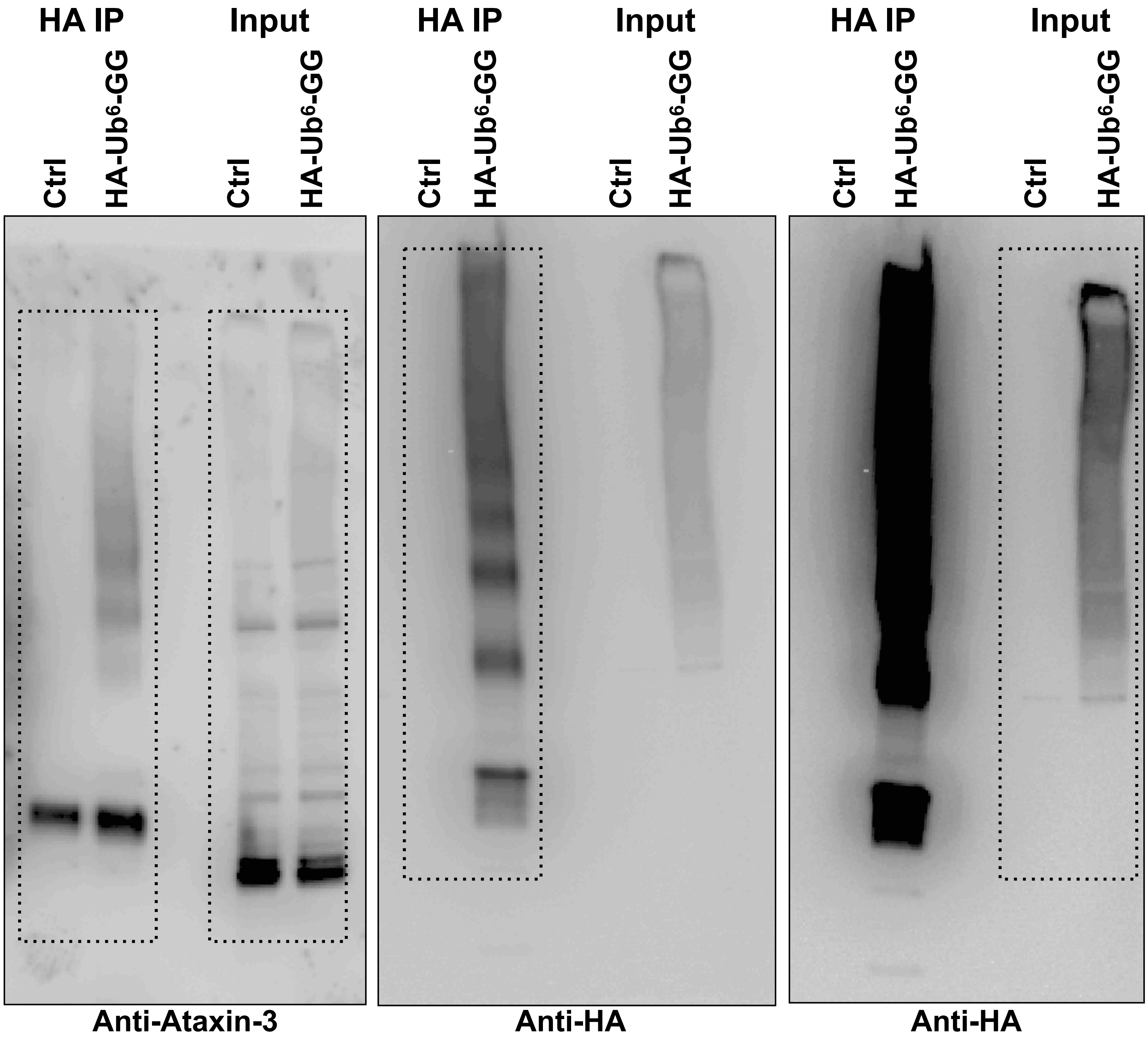

Two different exposures are shown of the same membrane for middle and right images because input and IP lanes were chosen from these blots for the main figures. Exposures: shorter, longer.

6D - Right portion

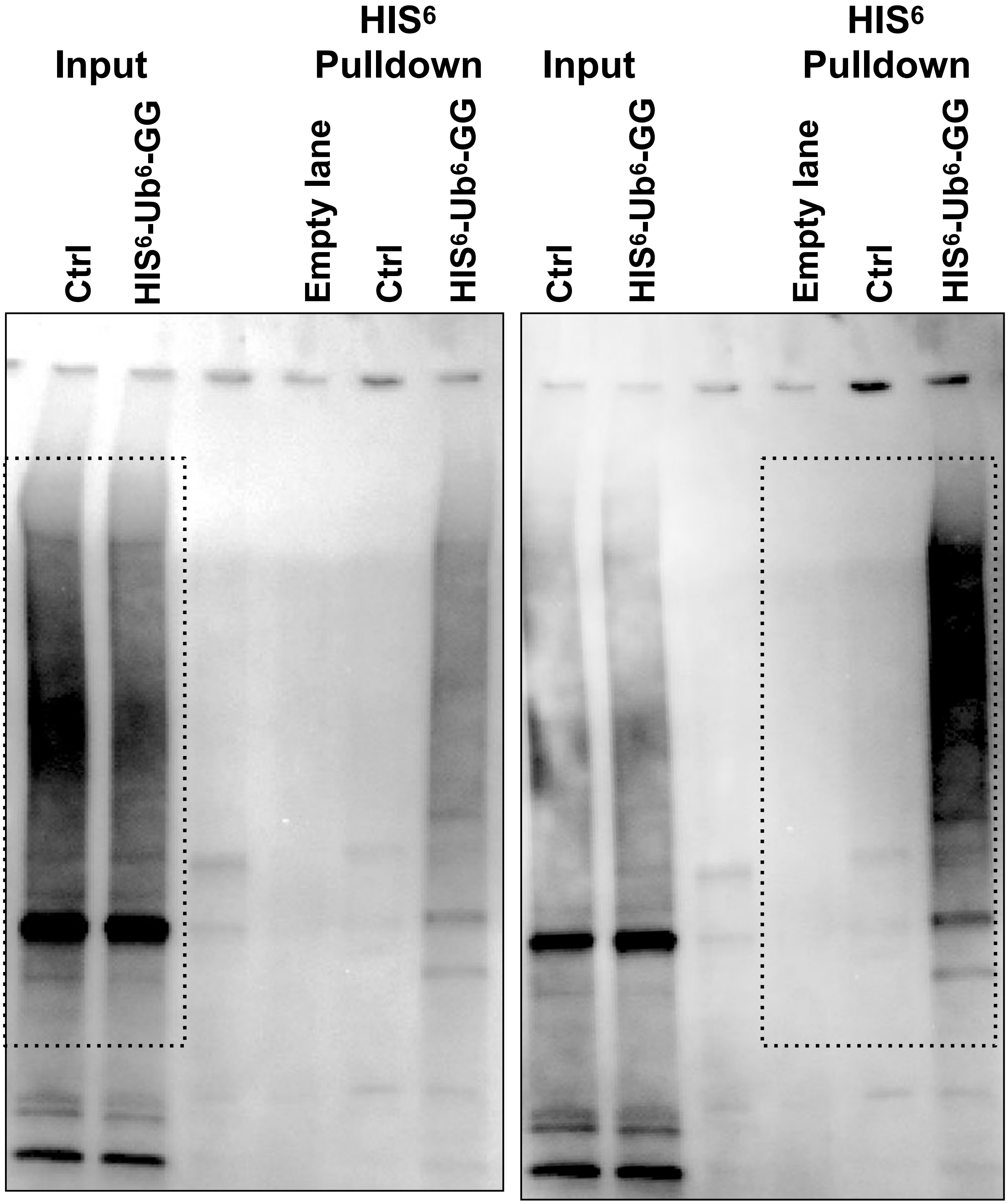

Anti-Ub (Total Ub)

Two different exposures are shown of the same membrane because input and IP lanes were chosen from these blots for the main figures. Exposures: shorter, longer. Signal for left portion of longer exposure was fading at the end.

Boxes: areas in main figure
